# Chemoresistance of *TP53* mutant acute myeloid leukemia requires the mevalonate byproduct, geranylgeranyl pyrophosphate, for induction of an adaptive stress response

**DOI:** 10.1038/s41375-025-02668-6

**Published:** 2025-07-09

**Authors:** Sarah J. Skuli, A’ishah Bakayoko, Marisa Kruidenier, Bryan Manning, Paige Pammer, Akmal Salimov, Owen Riley, Gisela Brake-Sillá, Derrick Dopkin, Michael Bowman, Leslie N. Martinez-Gutierrez, Colin C. Anderson, Julie A. Reisz, Roberta Buono, Madhuri Paul, Estelle Saland, Francesca Liccardo, Ann DeVine, Sarah Wong, Jimmy P. Xu, Eva Nee, Ryan Hausler, Steffen Boettcher, Said M. Sebti, Catherine Lai, Kara N. Maxwell, Jean-Emmanuel Sarry, David A. Fruman, Angelo D’Alessandro, Clementina Mesaros, Brian Keith, M. Celeste Simon, Pamela J. Sung, Gerald Wertheim, Nicolas Skuli, Robert L. Bowman, Andrew Matthews, Martin Carroll

**Affiliations:** 1 Division of Hematology & Oncology, Department of Medicine, University of Pennsylvania, Philadelphia, PA USA; 2 Immunology, Microenvironment & Metastasis Program, Ellen and Ronald Caplan Cancer Center, Wistar Institute, Philadelphia, PA USA; 3 Department of Cancer Biology, University of Pennsylvania, Philadelphia, PA USA; 4 Department of Medicine – Leukemia, Roswell Park Comprehensive Cancer Center, Buffalo, NY USA; 5 Department of Pharmacology and Therapeutics, Roswell Park Comprehensive Cancer Center, Buffalo, NY USA; 6 Department of Biochemistry and Molecular Genetics, University of Colorado Anschutz Medical Campus, Aurora, CO USA; 7 Department of Molecular Biology and Biochemistry, University of California, Irvine, CA USA; 8 Centre de Recherches en Cancérologie de Toulouse, Université de Toulouse, Toulouse, France; 9 Department of Research and Development, Tevogen Bio, Philadelphia, PA USA; 10 Department of Pharmacology, The University of Pennsylvania, Philadelphia, PA USA; 11 Department of Medical Oncology and Hematology, University of Zurich and University Hospital Zurich, Zurich, Switzerland; 12 Department of Pharmacology and Toxicology and Massey Comprehensive Cancer Center, Virginia Commonwealth University, Richmond, VA USA; 13 Abramson Family Cancer Research Institute, University of Pennsylvania Perelman School of Medicine, Philadelphia, PA USA; 14 Corporal Michael Crescenz Veterans Affairs Medical Center, Philadelphia, PA USA; 15 LabEx Toucan, Toulouse, France; 16 Équipe Labellisée Ligue Nationale Contre le Cancer, Toulouse, France; 17 Department of Cell and Developmental Biology, University of Pennsylvania, Philadelphia, PA USA; 18 Department of Pathology and Laboratory Medicine, Children’s Hospital of Philadelphia, Philadelphia, PA USA; 19 Department of Radiology, University of Pennsylvania, Philadelphia, PA USA

**Keywords:** Cancer metabolism, Cancer therapeutic resistance

## Abstract

Acute myeloid leukemia with mutations in *TP53* (*TP53*^mut^ AML) is fatal with a median survival of 6 months. RNA sequencing on purified AML patient samples showed that *TP53*^mut^ AML had higher expression of mevalonate pathway genes. Using novel, isogenic *TP53*^mut^ AML cell lines and primary samples, we determined that *TP53*^mut^ AML resistance to AML chemotherapy cytarabine (AraC) correlated with increased mevalonate pathway activity, a lower induction of reactive oxygen species (ROS), and a mitochondrial response with increased mitochondrial mass and oxidative phosphorylation. Pretreatment with the statin class of mevalonate pathway inhibitors reversed these effects and chemosensitized *TP53*^mut^ AML. The geranylgeranyl pyrophosphate (GGPP) branch of the mevalonate pathway was required for *TP53*^mut^ AML chemoresistance. In addition to its role in mitochondria biogenesis, we identified a novel function of GGPP in regulating glutathione for management of AraC-induced ROS. However, statins alone were inadequate to fully reverse chemoresistance in vivo and in a retrospective study of 364 *TP53*^mut^ AML patients who received chemotherapy concurrently with a statin. Finally, we identified clinical settings and strategies to successfully target the mevalonate pathway, particularly to address the unmet need of *TP53*^mut^ AML.

## Introduction

*TP53* mutant acute myeloid leukemia (*TP53*^mut^ AML) is the paramount clinical challenge in the field of leukemia. For *TP53*^mut^ AML patients, particularly those with bi-allelic alterations, standard therapies, including cytarabine (AraC)-based regimens as well as hypomethylating (HMA) agents with or without the BCL2 inhibitor venetoclax (Ven) all lead to a similar and dismal median overall survival of 6–9 months [1–4]. Due to this inherent therapy resistance, international guidelines recommend a clinical trial as the first approach to treatment of *TP53*^mut^ AML [5].

Our group and others have demonstrated that AML requires enhanced mitochondrial oxidative phosphorylation (OXPHOS) to survive chemotherapy, including AraC and Ven [6–10]. The correlation between chemoresistance and increased mitochondrial activity is especially interesting in the context of *TP53*^mut^ AML as loss of p53 leads to increased glycolysis and OXPHOS in both solid tumors and a recent AML model [11–13]. However, the relationship between increased mitochondrial activity and chemoresistance specifically in *TP53*^mut^ AML has yet to be addressed. Moreover, direct targeting of mitochondrial metabolism to reverse chemoresistance has been limited by toxicity due to essential mitochondrial functions in non-malignant cells [14]. Novel strategies are needed to identify and target pathways that contribute to mitochondrial metabolism primarily and specifically in cancer cells.

Here, we present transcriptional analysis of human AML samples demonstrating that mevalonate pathway genes are overexpressed in *TP53*^mut^ compared to more chemosensitive *TP53* wildtype (*TP53*^WT^) AML samples. The mevalonate pathway is a complex metabolic pathway that branches at the level of farnesyl pyrophosphate (FPP), forming isoprenoids like geranylgeranyl pyrophosphate (GGPP) or other cholesterol derivatives. Importantly, there is interdependence between the mevalonate pathway and mitochondrial metabolism [15, 16]. The mevalonate pathway is also necessary for tumorigenesis and metastatic potential in multiple *TP53*^mut^ solid tumor models [17–21]. In AML, several studies have implicated the mevalonate pathway in therapy resistance [22–26]. Early phase clinical trials investigating the addition of statins to AraC-based induction therapy showed a promising benefit in patients with relapsed/refractory, poor risk AML [27–29] but did not lead to statin incorporation into standard therapy. Notably, *TP53*^mut^ AML patients should be enriched in this population, but sequencing of *TP53* was not routinely performed at the time. Altogether, the role of p53 in AML metabolism and chemoresistance remains poorly understood.

We hypothesized that the mevalonate pathway is required for mitochondria-dependent chemoresistance in *TP53*^mut^ AML. We addressed this hypothesis with multiple orthogonal approaches, including previously unpublished, multi-institutional, retrospective clinical data, novel, isogenic *TP53*^mut^ AML cell lines and primary *TP53*^mut^ AML patient samples. Our studies indicate that the mevalonate pathway should be targeted in chemotherapy resistant *TP53*^mut^ AML.

## Methods

Additional methods provided in the supplement.

### Cell culture

Human AML cell lines (Supplementary Table 1E) were maintained in minimum essential medium-alpha (Gibco) supplemented with 10% fetal bovine serum (Hyclone). The cultured cells were split every 2-3 days and maintained in an exponential growth phase. Authenticated MOLM14 and HL60 cells were obtained from DSMZ and the liquid nitrogen stock was renewed every 2 years. MOLM13-*TP53* isogenic AML cell lines were generously provided by Dr. Stefen Boettcher and generated as previously described [30]. Cell lines were routinely tested for mycoplasma contamination.

### Primary AML specimens

Mononuclear cells (MNCs) from patients with AML were obtained from the Stem Cell and Xenograft Core (SCXC, IRB Protocol Number: 703185) at The University of Pennsylvania (UPenn). All samples were acquired through the SCXC following informed consent on the institutional review board-approved protocol. Samples were collected in accordance with federal and institutional guidelines and provided in a pathologically annotated and de-identified fashion. Sample sizes were chosen based on availability.

### Statistics

Statistical analysis was performed in Prism10 using unpaired two-sided Student’s *T* test, ANOVA, and two-sided Fisher’s exact test, as appropriate for each experiment and indicated in the legends. Multiple test correction was implemented using the Benjamini–Hochberg/FDR approach or the Bonferroni’s multiple comparisons test as indicated in the legends. For multiple comparisons, a range of significant adjusted *p* values, or FDR values, have been provided. The number of independent, biological replicates performed in the laboratory and used for statistical calculations are noted in the legends. The number of replicates for in vitro cell line assays was at least 3 to allow for statistical analysis. Asterisks denote statistical significance as described in figure legends. Error bars represent standard deviations.

## Results

### Primary *TP53*^mut^ AML RNA sequencing show upregulation of a cholesterol synthesis gene signature

**Fig. 1:**
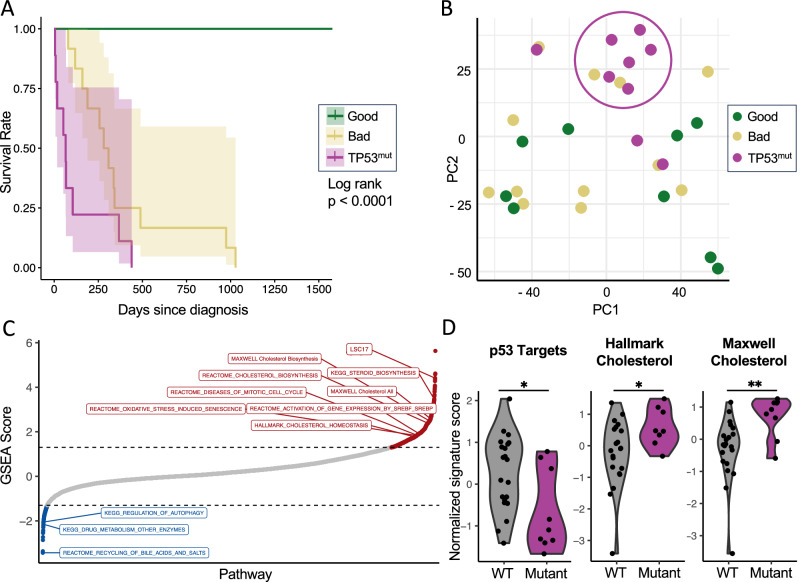
Untreated, primary *TP53*^mut^ AML are enriched for cholesterol synthesis gene expression. RNA sequencing was performed on highly purified, flow-sorted blasts of primary human AML diagnostic samples from patients with *TP53*^mut^ (*n* = 9), *TP53*^WT^ “bad” outcome AML (*n* = 12), and *TP53*^WT^ “good” outcome AML (*n* = 9). **A** Kaplan–Meier survival outcomes with statistical analysis by log-rank test. **B** principal component analysis with principal component 1 (PC1; 48.9%) and PC2 (15%), **C** GSEA scores from the Hallmark and other denoted gene sets, and **D** single sample GSEA with calculation of normalized signature scores for “TP53 Targets,” “Hallmark Cholesterol,” and “Maxwell Cholesterol.” Statistical analysis by Student’s *T* Test. *p*-values: * = <0.05, ** = <0.01, *** = <0.001.

To elucidate biological differences between *TP53*^mut^ and *TP53*^WT^ AML, we performed RNA sequencing on untreated primary AML blasts. We obtained 9 bi-allelic *TP53*^mut^ AML patient samples as well as 21 *TP53*^WT^ AML patient samples with either a “good” overall survival (OS) (*n* = 9; median OS not reached) or a “bad” OS (*n* = 12, median OS 294 days) (Fig. 1A, Supplementary Table 1A). The median OS of the *TP53*^mut^ AML patients was poor at 65 days (Fig. 1A; log-rank *p* < 0.0001 *versus TP53*^WT^ “good” or “bad” outcome patients). Principal component analysis suggested that the *TP53*^mut^ AML patient samples shared transcriptional similarity whereas the *TP53*^WT^ AML patient samples were more heterogeneous (Fig. 1B; PC1 48.9% and PC2 15%). Single sample GSEA (ssGSEA) demonstrated that primary *TP53*^mut^ compared to all *TP53*^WT^ AML had lower expression of *TP53* targets [31] (Fig. 1C, D, Supplementary Table 1B–D). Notably, multiple gene sets involved in cholesterol synthesis were upregulated in *TP53*^mut^AML (Fig. 1C, D, Supplementary Table 1C, D). These signatures were recapitulated in a cohort of primary *TP53*^mut^ (*n* = 17) *versus TP53*^WT^ (*n* = 87) AML samples identified in the BeatAML [32] dataset (Supplementary Fig. 1B–D).

### *TP53*^mut^ AML cell lines require the mevalonate pathway to survive AML chemotherapy, AraC

**Fig. 2:**
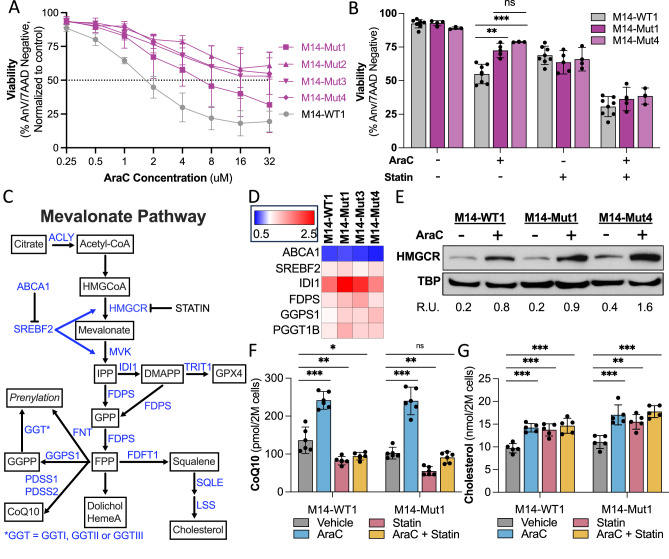
Chemoresistant *TP53*^mut^ AML cell lines upregulate the mevalonate pathway in response to AraC. **A** Cell viability of isogenic *TP53*^mut^ AML clones treated with increasing concentrations of AraC for 24 h and assessed by flow cytometry following staining with AnnexinV and 7AAD (*n* = 3). **B** Cell viability of representative isogenic *TP53*^mut^ and *TP53*^WT^ AML clones pretreated with 24 h of rosuvastatin (50 μM) followed by an additional 24 h of AraC (1μM) and assessed by flow cytometry following staining with AnnexinV and 7AAD (*n* = 7). Statistical analysis by one-way ANOVA with false discovery rate (FDR) correction for multiple comparisons. **C** Schema of the mevalonate pathway with key enzymes highlighted in blue and byproducts in squares. Subunits: FNT = FNTA & FNTB, GGTI = FNTA & PGGT1B, GGTII = RABGGTA & RABGGTB, GGTIII = PTAR1. **D** Gene expression of select mevalonate pathway genes in isogenic *TP53*^mut^ and *TP53*^WT^ AML clones following 24 h of AraC (1 μM) treatment, normalized to GAPDH, and compared to the vehicle for each clone as measured by qRT-PCR (*n* = 3). **E** Protein expression of HMGCR normalized to total binding protein (TBP) in isogenic *TP53*^mut^ and *TP53*^WT^ AML clones treated with vehicle or 18 h of AraC (1 μM), performed by western blot and quantified by imageJ as presented in relative units (R.U.) of HMGCR to TBP. **F** CoQ10 as pmol per 2 million cells and **G** cholesterol as nmol per 2 million cells in the M14-WT1 and M14-Mut1 clones in the same conditions as (**B**) and quantified by LC-HRMS (*n* = 5). Statistical analysis for (**F**, **G**) by two-way ANOVA with FDR correction for multiple comparisons. Adjusted *p*-values: * = <0.05, ** = <0.01, *** = <0.001. *n* is the number of replicates.

Isogenic *TP53*^mut^ AML cell line clones with complete loss (M14-Mut1, M14-Mut3) or missense-like mutations (M14-Mut2, M14-Mut4) of the p53 protein were developed using CRISPR/Cas9 editing of the AML cell line, MOLM14, with published guides [33, 34] and single cell cloning *via* serial dilution. The *TP53*^mut^ AML clones had consistently higher cell viability in response to AraC than the *TP53*^WT^ AML clone, M14-WT1, indicating that the *TP53*^mut^ AML clones were more chemoresistant (Fig. 2A). Furthermore, only the *TP53*^WT^ clones exhibited a rapid and significant increase in downstream p53 target, p21, at 6 and 24 h following AraC treatment or 6 h after irradiation (Supplementary Fig. 2A, B). To determine the impact of inhibition of the mevalonate pathway on chemoresistance, we pretreated the isogenic clones with the mevalonate pathway inhibitor, rosuvastatin, for 24 h prior to adding AraC. Cell death induced by AraC was augmented by rosuvastatin pre-treatment in all *TP53*^mut^ and *TP53*^WT^ AML clones (Fig. 2B, Supplementary Fig. 2C). Importantly, rosuvastatin chemosensitized the *TP53*^mut^ MOLM14 AML clones to AraC now to the same level as the *TP53*^WT^ clones (Fig. 2B, Supplementary Fig. 2C). We validated these findings in isogenic *TP53*^mut^ and *TP53*^WT^ MOLM13 AML clones [30, 35] (Supplementary Fig. 2D) and the HL60 AML cell line with parental homozygous loss of p53 (Supplementary Fig. 2E). XTT viability assays showed that the combination of AraC and rosuvastatin was synergistic in the isogenic MOLM14 clones using the SynergyFinder tool [36] (Supplementary Fig. 2F). Statin-induced chemosensitization is a class-effect as pitavastatin also chemosensitized the isogenic MOLM14 AML clones to AraC (Supplementary Fig. 2G) at concentrations achievable in patients [22, 37].

### Chemoresistant *TP53*^mut^ AML cell lines upregulate the mevalonate pathway in response to AraC

To study the mechanism by which statins chemosensitize *TP53*^mut^ AML, we first addressed the impact of p53 loss on the mevalonate pathway (Fig. 2C) at baseline and in response to AraC. Mevalonate pathway gene expression was assessed by qRT-PCR in representative *TP53*^mut^ and *TP53*^WT^ MOLM14 AML clones. AraC induced a time-dependent upregulation of multiple genes in the mevalonate pathway as well as the pathway’s primary transcription factor, *SREBF2*, in both *TP53*^mut^ and *TP53*^WT^ AML clones (Fig. 2D, Supplementary Fig. 2H for time course). The rate-limiting enzyme of the mevalonate pathway and the target of statins, HMGCR, is also regulated at the post-translational level [38, 39]. HMGCR protein expression was significantly increased by AraC in representative clones and highest in those with *TP53* mutations (Fig. 2E, Supplementary Fig. 2I). We also detected a significant increase in mevalonate byproducts, CoQ10 and cholesterol, in AraC-treated isogenic clones (Fig. 2F, G, respectively). Rosuvastatin pretreatment abrogated the AraC-induced increase in CoQ10 [Fig. 2F; M14-WT1: F(1,16) = 22, *p* < 0.001; M14-Mut1: F(1,16) = 26, *p* < 0.001], while rosuvastatin pretreatment alone or in combination with AraC was associated with a significant increase in cholesterol potentially *via* uptake (Fig. 2G).

### Increased OXPHOS and regulation of ROS correlate with AraC resistance in *TP53*^mut^ AML cell lines

**Fig. 3:**
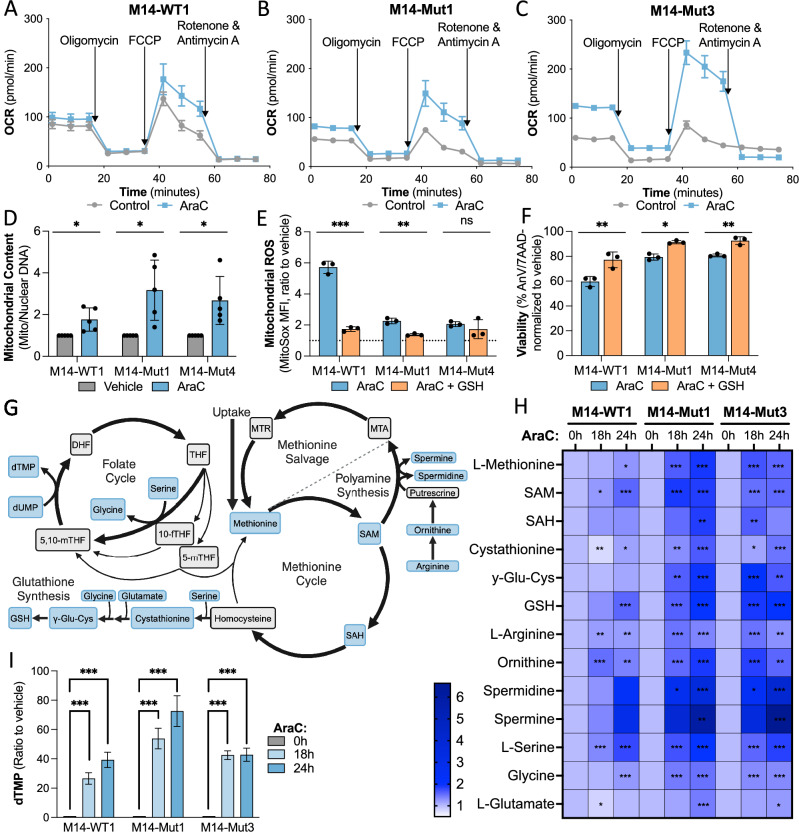
*TP53*^mut^ AML AraC resistance is associated with increased OXPHOS, ROS regulation and one-carbon metabolism. Oxygen consumption in pmol per minute in **A** M14-WT1, **B** M14-Mut1, and **C** M14-Mut3 treated with vehicle or AraC (1 μM) for 24 h and measured by Seahorse technology (*n* = 7, *n* = 8, and *n* = 8, respectively). AraC *versus* vehicle fold-changes: Basal OXPHOS of 1.2 (*p* = 0.05) for M14-WT1, 1.5 (*p* < 0.001) for M14-Mut1, 2.1 (*p* = 0.002) for M14-Mut3; Maximum-uncoupler-induced OXPHOS of 1.3 (*p* = 0.04) for M14-WT1, 2 (*p* < 0.001) for M14-Mut1, 2.7 (*p* < 0.001) for M14-Mut3. **D** Mitochondrial content presented as the ratio of mitochondrial to nuclear DNA measured by PCR in representative isogenic *TP53*^mut^ and *TP53*^WT^ AML clones treated with vehicle or AraC (1 μM) for 24 h. **E** Mitochondrial ROS presented as the ratio to vehicle of mean fluorescence intensity (MFI) of MitoSox in viable cells [determined by Fixable Viability Stain Red (FVS-R)] as measured by flow cytometry in representative isogenic *TP53*^mut^ and *TP53*^WT^ AML clones treated with AraC (1 μM, 24 h), glutathione (10 mM, 36 h), the combination, or their respective vehicles (*n* = 3). **F** Viability of cells normalized to respective vehicles treated as per (**E**) and measured by flow cytometry after staining with AnnexinV and 7AAD. **G** Schema of one-carbon metabolism with bold arrows highlighting the observed pathway increases in AraC-treated cells, particularly in *TP53*^mut^ AML clones. **H** Metabolomics in M14-WT1, M14-Mut1, and M14-Mut3 AML cell lines treated with vehicle, 18 h AraC (1 μM), or 24 h AraC (1 μM) (*n* = 5 per condition) presented as the ratio to respective cell line vehicle. **I** Relative abundance of dTMP for each condition relative to vehicle (*n* = 5). Statistical analysis by Student’s *T* test for (**D**–**F**), Z-tests for (**H**), and two-way ANOVA with Bonferroni’s multiple comparisons test for (**I**). Adjusted *p*-values: * = <0.05, ** = <0.01, *** = <0.001. *n* is the number of replicates.

Our group and others have demonstrated that AML AraC resistance correlates with increased OXPHOS. However, the impact of *TP53* mutations on mitochondrial metabolism in AML is not well understood. We performed a series of functional and structural assays to characterize mitochondria in our isogenic MOLM14 AML clones and *TP53*^mut^ HL60 cells. In response to AraC, only *TP53*^mut^ AML cells induced a significant increase in basal and maximum uncoupler-induced oxygen consumption as assessed by Seahorse (Fig. 3A–C, Supplementary Fig. 3A–J). The increase in OXPHOS was associated with a significant increase in mitochondrial mass as determined by measuring the ratio of mitochondrial to nuclear DNA by PCR in isogenic MOLM14 AML clones (Fig. 3D). We next assessed mitochondrial reactive oxygen species (ROS). Treatment of M14-WT1 with AraC was associated with a significant increase in ROS (6-fold increase, *p* < 0.001) (Fig. 3E). Correlating with chemoresistance, M14-Mut1 and M14-Mut4 exhibited a significantly lower induction of ROS in response to AraC (2-fold increases, *p* < 0.01 for M14-Mut1 and *p* = 0.4 for M14-Mut4) (Fig. 3E). Thus, there was a paradoxical improvement in ROS management in *TP53*^mut^ AML clones despite a significant increase in OXPHOS in response to AraC (Fig. 3A–C, E). Furthermore, AraC-induced ROS and cell viability was at least partially rescued by pretreatment with the antioxidant, reduced glutathione (GSH, Fig. 3E, F), suggesting that AraC-induced ROS directly correlates to induction of cell death.

### Metabolomics correlate *TP53*^mut^ AML induction of one-carbon metabolism to management of AraC-induced ROS

With the significant differences in AraC-induced OXPHOS and ROS between *TP53*^WT^ and *TP53*^mut^ AML cell lines, we next performed metabolomics in representative isogenic clones treated with AraC (Fig. 3G–I, Supplementary Fig. 3K, Supplementary Table 1F). *TP53*^mut^ AML AraC-treated cells exhibited striking alterations in one-carbon metabolism (Fig. 3G–I, Supplementary Fig. 3K). AraC induced a significant increase in methionine and downstream S-adenosylmethionine (SAM) (Fig. 3H). Only the *TP53*^mut^ AML clones increased SAM conversion to S-adenosylhomocysteine (SAH), which was associated with an increase in downstream cystathionine, γ-glutamyl-cysteine (γ-Glu-Cys), and GSH (Fig. 3H). GSH is a highly abundant essential cofactor for the enzyme-based glutathione system [40]. Polyamines are also an important ROS scavenger [41–43]. AraC was associated with a significant increase in the polyamine precursors arginine and ornithine (Fig. 3H). Only in *TP53*^mut^ AML clones did AraC induce a significant increase in the SAM-dependent polyamines, spermine and spermidine (Fig. 3H). One of the most significantly increased metabolites in response to AraC was deoxythymidine monophosphate (dTMP; Fig. 3I) and its precursors (Supplementary Fig. 3K), which may reflect an increase in the folic acid cycle. Altogether, this data suggests *TP53*^mut^ AML-specific induction of one-carbon metabolism, particularly glutathione and polyamines synthesis, for AraC resistance.

### AraC-induced alterations in OXPHOS and glutathione synthesis are mevalonate pathway-dependent

**Fig. 4:**
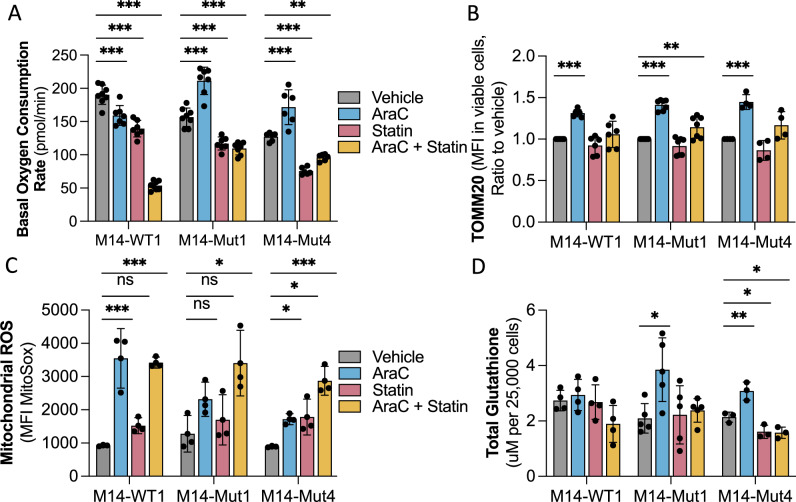
OXPHOS and ROS regulation in response to AraC are mevalonate-pathway dependent. For (**A**–**D**), representative isogenic *TP53*^mut^ and *TP53*^WT^ AML clones were treated with rosuvastatin (50 μM) for a total of 48 h with AraC (1uM) added for the last 24 h and the following experiments subsequently performed: **A** Basal oxygen consumption rate in pmol per minute assessed by Seahorse technology (*n* = 7), **B** TOMM20 presented as MFI in viable cells (by FVS-R) assessed by flow cytometry (*n* = 6, *n* = 7, *n* = 4 for respective cell lines), **C** mitochondrial ROS presented as the ratio to vehicle of MFI of MitoSox in viable cells (by FVS-R) as measured by flow cytometry (*n* = 3), and **D** total glutathione presented as μM per 25,000 cells (*n* = 4, *n* = 5, *n* = 3 for respective cell lines). Statistical analysis by two-way ANOVA with FDR correction for multiple comparisons. Adjusted *p*-values: * = <0.05, ** = <0.01, *** = <0.001. *n* is the number of replicates.

Multiple mevalonate pathway byproducts are important for mitochondrial metabolism and redox management. Thus, we next assessed the impact of rosuvastatin on mitochondrial and redox parameters in representative *TP53*^mut^ and *TP53*^WT^ MOLM14 AML clones and *TP53*^mut^ HL60 cells. Rosuvastatin alone decreased basal and max OXPHOS and completely abrogated the AraC-induced increase in OXPHOS (Fig. 4A, Supplementary Fig. 4A–H). We then evaluated intracellular levels of TOMM20, a mitochondria-specific protein that is a surrogate for mitochondrial mass (Fig. 4B), and mitochondrial ROS (Fig. 4C). Cells treated with single agent rosuvastatin trended towards decreased TOMM20 (Fig. 4B) and increased mitochondrial ROS (Fig. 4C). Importantly, rosuvastatin pretreatment in *TP53*^mut^ MOLM14 AML cells completely abrogated the AraC-induced increase in TOMM20 while significantly increasing mitochondrial ROS to the same degree as M14-WT1 (Fig. 4B, C). We then assessed total glutathione and the ratio of reduced to oxidized glutathione (i.e., GSH/GSSG). In correlation with improved ROS management in *TP53*^mut^ AML clones, M14-Mut1 and M14-Mut4 responded to AraC with a significant increase in total glutathione, including a specific increase in GSH, which were both abrogated by rosuvastatin pretreatment (Fig. 4D, Supplementary Fig. 4I). This data suggested that a mevalonate pathway-dependent mitochondrial response and ROS management was key for AraC chemoresistance in *TP53*^mut^ AML.

### AraC resistance in *TP53*^*m*ut^ AML requires the mevalonate pathway byproduct, GGPP

**Fig. 5:**
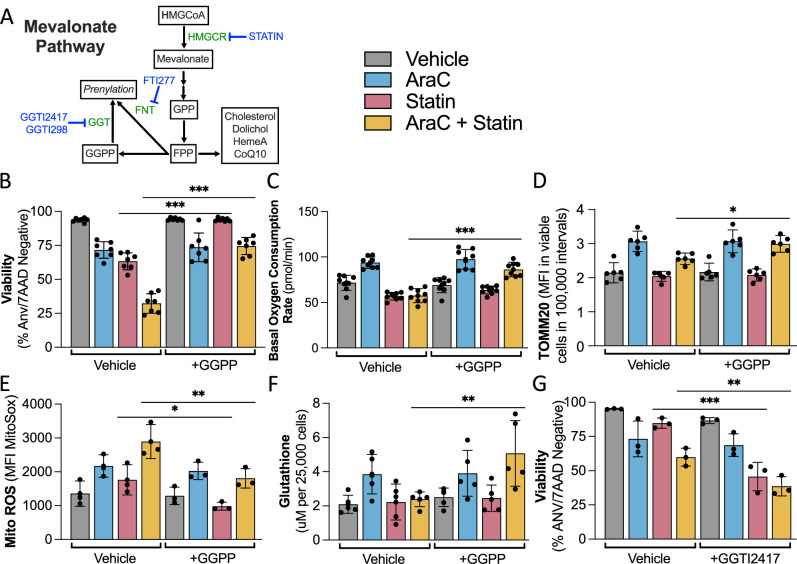
AraC resistance of *TP53*^*m*ut^ AML requires GGPP for ROS regulation and enhanced OXPHOS. **A** Abridged schema of latter half of the mevalonate pathway with essential genes in green and known chemical inhibitors in blue. All experiments in Fig. 6B-I were performed in the M14-Mut1 clone pretreated for 24 h with rosuvastatin (50 μM) and/or either vehicle, GGPP (1 μM) or GGTI2417 (4 μM) followed by an additional 24 h with vehicle or AraC (1 μM) with the subsequent assessment of **B** cell viability presented as the percentage of annexinV and 7AAD negative cells by flow cytometry with or without GGPP (*n* = 7), **C** basal oxygen consumption rate in pmol/minute as assessed by Seahorse technology with or without GGPP (*n* = 8), **D** TOMM20 presented as MFI in viable cells (by FVS-R) assessed by flow cytometry with or without GGPP (*n* = 5), **E** mitochondrial ROS presented as the MFI of MitoSox in viable cells (by FVS-R) as measured by flow cytometry with or without GGPP (*n* = 3), **F** total glutathione presented as μM per 25,000 cells with or without GGPP (*n* = 5), and **G** cell viability presented as the percentage of annexinV and 7AAD negative cells by flow cytometry with or without GGTI2417 (*n* = 3). Statistical analysis by three-way ANOVA with FDR correction for multiple comparisons, including comparison of a treatment with or without the rescue agent or additional inhibitor (i.e., AraC + Statin *vs* AraC + Statin + GGPP). Adjusted *p*-values: * = <0.05, ** = <0.01, *** = <0.001. *n* is the number of replicates.

To determine the specific mevalonate pathway byproducts necessary for mitochondria-dependent chemoresistance, we performed rescue experiments in the representative *TP53*^mut^ AML clone, M14-Mut1, treated with a rescue agent and rosuvastatin for 48 h with AraC added for the last 24 h (Schema summarizing targets in Fig. 5A). Co-treatment of M14-Mut1 with mevalonate, which is produced by the direct statin target, HMGCR, completely rescued the effect of rosuvastatin on cell viability and OXPHOS (Supplementary Fig. 5A–D). Subsequently, co-treatment of M14-Mut1 with downstream GGPP also rescued the effects of a statin on cell viability (Fig. 5B), OXPHOS (Fig. 5C, Supplementary Fig. 5E, F), TOMM20 as a mitochondria mass surrogate (Fig. 5D), mitochondrial ROS (Fig. 5E), and total and reduced glutathione (Fig. 5F, Supplementary Fig. 5G). GGPP did not rescue the rosuvastatin-induced decrease in coenzyme Q10 (CoQ10) in either M14-Mut1 or M14-WT1 (Supplementary Fig. 5H). These data indicated that rosuvastatin’s activity is specific to the mevalonate pathway, dependent on the GGPP branch of the pathway, and independent of the cholesterol and CoQ10 branches. The presence of GGPP correlated with improved glutathione synthesis and reduced ROS, specifically in the M14-Mut1 clone (M14-WT1 experiments in Supplementary Fig. 6). Finally, we validated that specific geranylgeranyl transferase (GGT) inhibitors, GGTI2417 [44, 45] and GGTI298 [46–48], that block the incorporation of GGPP onto targets, but not the farnesyltransferase (FNT) inhibitor, FTI277, recapitulated rosuvastatin’s impact on cell viability alone or in combination with AraC (Fig. 5G, Supplementary Figs. 5I–K,  6M–O).

### In vitro statin pretreatment fully abrogates OXPHOS and chemoresistance in primary *TP53*^mut^ AML

**Fig. 6:**
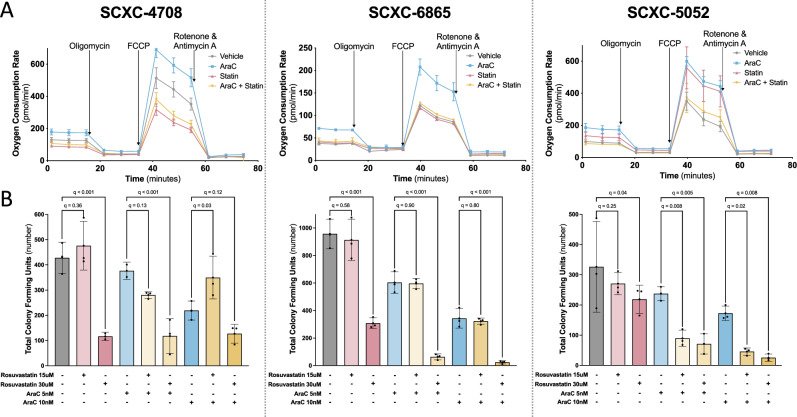
In vitro statin pretreatment fully abrogates OXPHOS and chemoresistance in primary *TP53*^mut^ AML*.* **A** Oxygen consumption rate (pmol/min) assessed by Seahorse technology in previously viably frozen primary *TP53*^mut^ AML samples that were resuspended in X-Vivo media with 20% BIT serum and 10 ng/mL of cytokines FLT3, SCF, IL3 and IL6, pretreated with rosuvastatin (50 μM) for 24 h followed by AraC (1 μM) treatment for an additional 24 h, and depleted of dead cells *via* the AnnexinV dead cell removal kit prior to plating for Seahorse analysis. Each sample (SCXC-4708, -6865, -5052) was performed once with 5 technical replicates per condition. AraC *vs* vehicle fold changes were as follows (basal and max OXPHOS, respectively): SCXC-4708 1.4-fold (*p* < 0.001) and 1.3-fold (*p* < 0.001); SCXC-6865 1.9-fold (*p* = 0.005) and 1.7-fold (*p* = 0.02); SCXC-5052 1.9-fold (*p* < 0.001) and 1.7-fold (*p* < 0.001). **B** Total number of colony forming units assessed 14 days after plating previously viably frozen primary *TP53*^mut^ AML samples (SCXC-4708, -6865, -5052) treated on day 0 with rosuvastatin (15 μM or 30 μM) and/or AraC (5 nM or 10 nM) with 3 replicates per condition. Statistical analysis by one-way ANOVA with FDR correction for multiple comparisons. *n* is the number of replicates.

We then evaluated the efficacy of the drug combination in four primary *TP53*^mut^ AML samples in vitro (annotations in Supplementary Table 1E). Three of the four samples demonstrated a significant increase in basal and max OXPHOS in response to AraC, which was blocked by rosuvastatin pretreatment (Fig. 6A). Those 3 samples also demonstrated a dose-dependent decrease in colony forming unit ability by the combination of AraC and rosuvastatin (Fig. 6B). Furthermore, the AraC-induced increase in OXPHOS correlated with an increase in mitochondrial mass as measured by flow cytometry after staining for mitochondrial protein TOMM20 in two of the three primary samples (Supplementary Fig. 7A). Of note, these three OXPHOS-inducing primary samples all exhibited bi-allelic *TP53* mutations without other AML-driver mutations (Supplementary Table 1E). The fourth *TP53*^mut^ AML sample, SCXC-7575, had co-mutations in *IDH2*, *ASXL1*, *KRAS* and *CEBPA* (Supplementary Table 1E) with recent studies correlating *IDH2* or *ASXL1* co-mutations with improved responses to therapy in *TP53*^mut^ AML^1^. SCXC-7575 did not demonstrate a significant increase in OXPHOS in response to AraC (Supplementary Fig. 7B), which correlated with a smaller benefit of combination treatment on CFUs (Supplementary Fig. 7C). These studies suggested that primary human *TP53*^mut^ AML cells required the mevalonate pathway to survive in vitro AraC treatment and that survival correlated with increased OXPHOS. Furthermore, the absence of an AraC-induced OXPHOS phenotype predicted higher sensitivity to AraC and a decreased benefit of rosuvastatin pretreatment.

### PDX modeling of *TP53*^mut^ AML reveals only partial reversal of AraC resistance by a statin

**Fig. 7:**
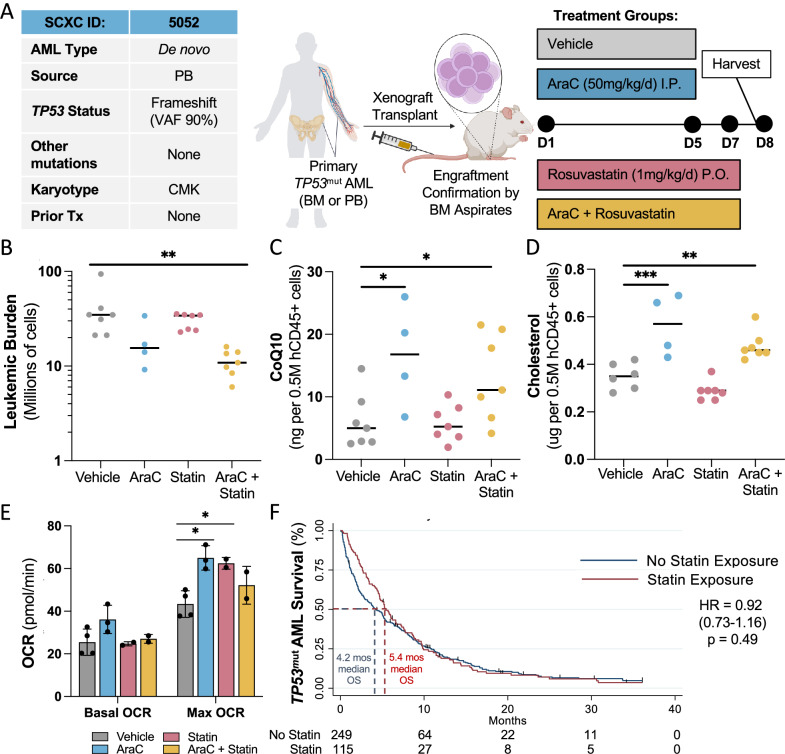
Statins are inadequate to fully reverse AraC resistance in PDX models and a retrospective cohort of *TP53*^mut^ AML patients. **A** Schema (created in BioRender) of *TP53*^mut^ AML PDX model. In brief, primary, human *TP53*^mut^ AML cells (chemoresistant SCXC-5052) were injected into the tail veins of busulfan-pretreated NSG mice. Engraftment was confirmed on bone marrow aspirates followed by initiation of treatment with vehicle, AraC (50 mg/kg/day IP for D1-5), rosuvastatin (1 mg/kg/day PO for D1-7) or the two drugs in combination. On D8 mice were humanely sacrificed and bilateral femurs/tibias and spleen were harvested (*n* = 7 mice per condition, early death of 3 mice in AraC condition). **B** Leukemic burden in millions of hCD45 + hCD33+ cells in the bone marrow and spleen combined for each mouse as quantified with counting beads by flow cytometry. **C** CoQ10 (ng) and **D** cholesterol (μg) per 0.5 million magnetic bead-purified hCD45+ leukemic cells from the bone marrow of mice with each circle representing the average of 1 mouse, as assessed by MS (5 replicates per mouse; *n* = 7, 4, 7, 7 mice for Control, AraC, Statin and AraC + Statin, respectively). **E** Basal and maximum coupler-induced oxygen consumption in pmol/minute assessed by Seahorse technology in magnetic bead-purified hCD45+ leukemic cells from the bone marrow with each circle representing the average of 1 mouse (*n* = 4, 3, 2, 2 mice for Control, AraC, Statin and AraC + Statin, respectively). **F** Kaplan–Meier survival estimates from a retrospective study of newly diagnosed *TP53*^mut^ AML patients (*n* = 364) who did or did not receive a concurrent statin during induction therapy (*n* = 115 and *n* = 249, respectively). Unless otherwise noted, statistical analysis by two-way ANOVA with FDR correction for multiple comparisons. Adjusted *p*-values: * = <0.05, ** = <0.01, *** = <0.001. *n* is the number of replicates. OCR oxygen consumption rate.

We established PDX mouse models of *TP53*^mut^ AML in NOD scid gamma^−/−^ (NSG) mice. Following engraftment of SCXC-5052, mice were treated with vehicle, AraC (50 mg/kg/day intraperitoneally on days 1–5) as routinely performed in the laboratory [6], rosuvastatin (1 mg/kg/day by oral gavage on days 1–7, the maximum dose tolerated in patients [49]) or the combination (schema in Fig. 7A). Mouse bone marrow (BM) and spleen were harvested on days 8–9 and leukemic burden was quantified. Only the drug combination led to a significant though modest decrease in leukemic burden (Fig. 7B). Surviving leukemic cells from AraC-treated mice had a significant increase in mevalonate pathway byproducts, CoQ10 (Fig. 7C) and cholesterol (Fig. 7D), and a significant increase in maximal OXPHOS with a trend towards increased basal OXPHOS (Fig. 7E, Supplementary Fig. 8A). However, rosuvastatin did not fully reverse AraC-induced mevalonate pathway byproduct accumulation or OXPHOS, suggesting inadequate inhibition of the mevalonate pathway by rosuvastatin in vivo (Fig. 7C–E). The experiment was also performed with the primary sample, SCXC-7575. Correlating with the in vitro data, AraC alone significantly reduced leukemic burden in the chemosensitive SCXC-7575 *TP53*^mut^ *IDH2*^*mut*^ *ASXL1*^*mut*^ *AML* PDX mouse model with no additional benefit from rosuvastatin [Supplementary Fig. 8B; F(1,22) = 0.5, *p* = 0.5]. Furthermore, AraC-persisting leukemic cells did not demonstrate increased basal or max OXPHOS (Supplementary Fig. 8C, D). Altogether, *TP53*^mut^ AML AraC resistance in vivo correlated with a mevalonate pathway-dependent increase in OXPHOS.

### In a retrospective analysis of *TP53*^mut^ AML patients undergoing induction therapy, co-administration of a statin is safe but does not improve survival

Statins are one of the most prescribed drugs for cardiovascular disease. Thus, a retrospective analysis was performed to determine the survival impact of concomitant statin with induction therapy in patients with newly diagnosed *TP53*^mut^ AML. Retrospective chart reviews were performed on 364 *TP53*^mut^ AML patients treated at one of two tertiary centers in the United States between 2013 and 2023 (Supplementary Table 1G). 32% of patients received a statin during induction therapy for *TP53*^mut^ AML. Statin-treated patients had more indications for a statin, such as a history of coronary artery disease and stroke (Fisher’s exact *p* < 0.001) but were otherwise well matched. An unadjusted Kaplan–Meier survival estimate suggested a non-significant 1.2-month improvement in median OS in patients who received a statin with induction therapy (Fig. 7F).

## Discussion

*TP53* mutations confer chemoresistance in AML, rendering standard treatments largely ineffective and leading to dismal patient outcomes [3, 4, 50]. Our work identifies metabolic drivers of AraC resistance in *TP53*^mut^ AML. We demonstrate for the first time that *TP53*^mut^ AML requires a mevalonate pathway-dependent adaptive stress response to survive chemotherapy. We provide compelling evidence that the GGPP branch of the pathway is crucial for chemoresistance through its regulation of (1) glutathione to manage chemotherapy-induced ROS and (2) mitochondrial mass and activity. We delve into mechanistic insights and therapeutic implications of targeting the mevalonate pathway in AML.

Our study has furthered our understanding of how cells resist AraC-induced cell death. Using novel isogenic *TP53*^mut^ MOLM14 AML cell lines, we observe that *TP53*^WT^ AML cells exhibit a significant increase in mitochondrial ROS in response to AraC. Conversely, *TP53*^mut^ AML has a higher antioxidant capacity and ability to regulate AraC-induced ROS that correlates with survival. We subsequently show that *TP53*^mut^ AML cells respond to AraC with significantly increased one-carbon metabolites directed towards synthesis of GSH and polyamines, which both play a role in cellular defense against ROS [40–43]. Notably, the upregulation of one-carbon metabolism is more robust in *TP53*^mut^ AML and only the *TP53*^mut^ AML clones increase levels of the polyamines, spermidine and spermine, in response to AraC. Interestingly, active wildtype p53 is known to inhibit the transcription of genes involved in polyamine synthesis (*ARG1, ODC1)* and promotes transcription of genes involved in polyamine catabolism (*SSAT)*, which then increases ROS [41]. Altogether, upregulation of one-carbon metabolism may be crucial for *TP53*^mut^ AML cells to survive AraC, particularly through production of antioxidants and subsequent ROS management.

Survival following AraC treatment is also associated with an increase in mitochondrial mass and OXPHOS, specifically in *TP53*^mut^ AML cell lines and primary samples in vitro and in vivo. A study evaluating the impact of *TP53* missense mutations in solid tumors found that stable endogenous *TP53* mutations led to *decreased* OXPHOS whereas inducible and transient *TP53*^mut^ models had *increased* OXPHOS [51]. Our data support these findings in which stable *TP53*^mut^ AML single cell clones at baseline have *decreased* OXPHOS (Fig. 3A–C, Supplementary Fig. 3A–J). However, when the cells are stressed with AraC, for example, we observe a significant *increase* in OXPHOS and mitochondrial mass similar to the inducible/transient *TP53*^mut^ solid tumor models. Thus, it is crucial to evaluate OXPHOS specifically in the context of stress in *TP53*^mut^ *versus TP53*^WT^ cancer models. Furthermore, the critical determinants of chemoresistance may be *both* the basal level of OXPHOS in AML cells and their ability to dynamically modify OXPHOS in response to stress.

Targeting metabolism, particularly direct mitochondrial inhibition, to reverse chemoresistance remains a challenge in the field [14]. Inhibiting the mevalonate pathway may be an indirect but effective way to overcome toxicity from direct mitochondrial inhibition. However, the optimal mevalonate pathway inhibitor may not be a statin. Combination rosuvastatin and AraC only modestly reduced *TP53*^mut^ AML leukemic burden in vivo, which correlated with partial abrogation of the mevalonate pathway and OXPHOS (Fig. 7B–E). Furthermore, while our retrospective analysis showed that a broad range of statins at different doses appear to be well tolerated and safe in combination with AML therapy in *TP53*^mut^ AML patients, there was no survival benefit (Fig. 7F). Statin anti-cancer efficacy in mouse and human models remains limited by (1) statin pharmacokinetics and pharmacodynamics that make it challenging to achieve doses in humans that inhibit geranylgeranylation [52, 53], which is further exacerbated in rodent models [54], (2) rescue of mevalonate pathway inhibition by dietary presence of mevalonate byproducts [55], and (3) highly conserved feedback loops that induce mevalonate pathway upregulation following inhibition [38]. Future preclinical studies should focus on robust mevalonate pathway inhibition in chemoresistant AML.

To optimize mevalonate pathway inhibition for anti-cancer benefit, it is essential to understand the specific role(s) of the mevalonate pathway in chemoresistance. We determined that GGPP is the essential byproduct required for AraC resistance. Other groups have validated the importance of this byproduct in multiple tumor models [21–23, 25, 56, 57]. However, the specific function of GGPP has remained elusive. GGPP may have multiple roles, such as regulation of GTPases that (1) play a role in mitochondrial biogenesis and (2) control glutathione levels. Notably, GGPP-dependency for maintaining glutathione has only been previously described in adipose tissue [58]. Future studies will define the specific mechanisms by which GGPP regulates mitochondrial biogenesis and glutathione synthesis and determine the therapeutic benefit of direct targeting of GGPP in *TP53*^mut^ AML. We anticipate that novel and specific GGT inhibitors, such as GGTI2418 which has shown significant promise in clinical trials for therapy-refractory peripheral T-cell lymphomas [59], will also be efficacious in *TP53*^mut^ AML.

## Supplementary information

Supplementary Materials

Supplementary Table 1

## Data Availability

Sequencing data available: GSE295181. Code available at https://github.com/bowmanr/Skuli_TP53_mevalonate .

## References

[1] Badar T, Nanaa A, Atallah E, Shallis RM, Craver EC, Li Z, et al. Prognostic impact of ‘multi-hit’ *versus* ‘single hit’ *TP53* alteration in patients with acute myeloid leukemia: results from the Consortium on Myeloid Malignancies and Neoplastic Diseases. Haematologica. 2024;109:3533–42. 10.3324/haematol.2024.285000 PMC11532685 38813716

[2] Grob T, Al Hinai ASA, Sanders MA, Kavelaars FG, Rijken M, Gradowska PL, et al. Molecular characterization of mutant *TP53*acute myeloid leukemia and high-risk myelodysplastic syndrome. Blood. 2022;139:2347–54. 10.1182/blood.2021014472 PMC11022827 35108372

[3] Bewersdorf JP, Shallis RM, Gowda L, Wei W, Hager K, Isufi I, et al. Clinical outcomes and characteristics of patients with TP53-mutated acute myeloid leukemia or myelodysplastic syndromes: a single center experience*. Leuk Lymphoma. 2020;61:2180–90. 32362171 10.1080/10428194.2020.1759051 PMC7603787

[4] Pasca S, Haldar SD, Ambinder A, Webster JA, Jain T, Dalton WB, et al. Outcome heterogeneity of TP53-mutated myeloid neoplasms and the role of allogeneic hematopoietic cell transplantation. Haematologica. 2024;109:948–52. 37731390 10.3324/haematol.2023.283886 PMC10905097

[5] Pollyea DA, Altman JK, Chair V, Assi R, Bachiashvili K, Raj Bhatt V, et al. NCCN guidelines version 2.2025 acute myeloid leukemia (age ≥18 years) continue NCCN guidelines panel disclosures ξ Bone marrow transplantation ‡ Hematology/Hematology oncology Þ Internal medicine † Medical oncology ≠ Pathology * Discussion Section Writing Committee Member. 2025. https://www.nccn.org/home/member-.

[6] Farge T, Saland E, de Toni F, Aroua N, Hosseini M, Perry R, et al. Chemotherapy-resistant human acute myeloid leukemia cells are not enriched for leukemic stem cells but require oxidative metabolism. Cancer Discov. 2017;7:716–35. 28416471 10.1158/2159-8290.CD-16-0441 PMC5501738

[7] Scotland S, Saland E, Skuli N, De Toni F, Boutzen H, Micklow E, et al. Mitochondrial energetic and AKT status mediate metabolic effects and apoptosis of metformin in human leukemic cells. Leukemia. 2013;27:2129–38. 23568147 10.1038/leu.2013.107 PMC10869165

[8] Bosc C, Saland E, Bousard A, Gadaud N, Sabatier M, Cognet G, et al. Mitochondrial inhibitors circumvent adaptive resistance to venetoclax and cytarabine combination therapy in acute myeloid leukemia. Nat Cancer. 2021;2:1204–23. 35122057 10.1038/s43018-021-00264-y

[9] Jones CL, Stevens BM, D'Alessandro A, Reisz JA, Culp-Hill R, Nemkov T, et al. Inhibition of amino acid metabolism selectively targets human leukemia stem cells. Cancer Cell. 2018;34:724–740.e4. 30423294 10.1016/j.ccell.2018.10.005 PMC6280965

[10] Stevens BM, Jones CL, Pollyea DA, Culp-Hill R, D’Alessandro A, Winters A, et al. Fatty acid metabolism underlies venetoclax resistance in acute myeloid leukemia stem cells. Nat Cancer. 2020;1:1176–87. 33884374 10.1038/s43018-020-00126-z PMC8054994

[11] Nechiporuk T, Kurtz SE, Nikolova O, Liu T, Jones CL, D'Alessandro A, et al. The TP53 apoptotic network is a primary mediator of resistance to BCL2 inhibition in AML cells. Cancer Discov. 2019;9:910–25. 31048320 10.1158/2159-8290.CD-19-0125 PMC6606338

[12] Zhang C, Liu J, Liang Y, Wu R, Zhao Y, Hong X, et al. Tumour-associated mutant p53 drives the Warburg effect. Nat Commun. 2013;4:2935 24343302 10.1038/ncomms3935 PMC3969270

[13] Basu S, Gnanapradeepan K, Barnoud T, Kung CP, Tavecchio M, Scott J, et al. Mutant p53 controls tumor metabolism and metastasis by regulating PGC-1α. Genes Dev. 2018;32:230–43. 29463573 10.1101/gad.309062.117 PMC5859965

[14] Yap TA, Daver N, Mahendra M, Zhang J, Kamiya-Matsuoka C, Meric-Bernstam F, et al. Complex I inhibitor of oxidative phosphorylation in advanced solid tumors and acute myeloid leukemia: phase I trials. Nat Med. 2023;29:115–26. 36658425 10.1038/s41591-022-02103-8 PMC11975418

[15] Tricarico P, Crovella S, Celsi F. Mevalonate pathway blockade, mitochondrial dysfunction and autophagy: a possible link. Int J Mol Sci. 2015;16:16067–84. 26184189 10.3390/ijms160716067 PMC4519939

[16] Ishihara N, Otera H, Oka T, Mihara K. Regulation and physiologic functions of GTPases in mitochondrial fusion and fission in Mammals. Antioxid Redox Signal. 2013;19:389–99. 22871170 10.1089/ars.2012.4830

[17] Freed-Pastor WA, Mizuno H, Zhao X, Langerød A, Moon SH, Rodriguez-Barrueco R, et al. Mutant p53 disrupts mammary tissue architecture via the mevalonate pathway. Cell. 2012;148:244–58. 22265415 10.1016/j.cell.2011.12.017 PMC3511889

[18] Moon SH, Huang CH, Houlihan SL, Regunath K, Freed-Pastor WA, Morris JP, et al. p53 represses the mevalonate pathway to mediate tumor suppression. Cell. 2019;176:564–580.e19. 30580964 10.1016/j.cell.2018.11.011 PMC6483089

[19] Oni TE, Biffi G, Baker LA, Hao Y, Tonelli C, Somerville TDD, et al. SOAT1 promotes mevalonate pathway dependency in pancreatic cancer. J Exper Med. 2020;217. 10.1084/jem.20192389 PMC7478739 32633781

[20] Kaymak I, Maier CR, Schmitz W, Campbell AD, Dankworth B, Ade CP, et al. Mevalonate pathway provides ubiquinone to maintain pyrimidine synthesis and survival in p189-203-deficient cancer cells exposed to metabolic stress. Cancer Res. 2020;80. 10.1158/0008-5472.CAN-19-0650 31744820

[21] Guo C, Wan R, He Y, Lin SH, Cao J, Qiu Y, et al. Therapeutic targeting of the mevalonate–geranylgeranyl diphosphate pathway with statins overcomes chemotherapy resistance in small cell lung cancer. Nat Cancer. 2022;3:614–28. 35449308 10.1038/s43018-022-00358-1

[22] Lee JS, Roberts A, Juarez D, Vo TTT, Bhatt S, Herzog L, et al. Statins enhance efficacy of venetoclax in blood cancers. Sci Transl Med. 2018;10. 10.1126/scitranslmed.aaq1240 PMC6336198 29899021

[23] Zhou C, Li J, Du J, Jiang X, Xu X, Liu Y, et al. HMGCS1 drives drug-resistance in acute myeloid leukemia through endoplasmic reticulum-UPR-mitochondria axis. Biomed Pharmacother. 2021;137:111378. 33601148 10.1016/j.biopha.2021.111378

[24] Pandyra A, Mullen PJ, Kalkat M, Yu R, Pong JT, Li Z, et al. Immediate utility of two approved agents to target both the metabolic mevalonate pathway and its restorative feedback loop. Cancer Res. 2014;74:4772–82. 24994712 10.1158/0008-5472.CAN-14-0130

[25] Xia Z, Tan M, Wei-Lynn Wong W, Dimitroulakos J, Minden M, Penn L. Blocking protein geranylgeranylation is essential for lovastatin-induced apoptosis of human acute myeloid leukemia cells. Leukemia. 2001;15:1398–407. 11516100 10.1038/sj.leu.2402196

[26] Mueller J, Schimmer RR, Koch C, Schneiter F, Fullin J, Lysenko V, et al. Targeting the mevalonate or Wnt pathways to overcome CAR T-cell resistance in TP53-mutant AML cells. EMBO Mol Med. 2024;16:445–74. 38355749 10.1038/s44321-024-00024-2 PMC10940689

[27] Kornblau SM, Banker DE, Stirewalt D, Shen D, Lemker E, Verstovsek S, et al. Blockade of adaptive defensive changes in cholesterol uptake and synthesis in AML by the addition of pravastatin to idarubicin + high-dose Ara-C: a phase 1 study. Blood. 2007;109:2999–3006. 17158228 10.1182/blood-2006-08-044446 PMC1852228

[28] Advani AS, Mcdonough S, Copelan E, Willman C, Mulford DA, List AF, et al. SWOG0919: a Phase 2 study of idarubicin and cytarabine in combination with pravastatin for relapsed acute myeloid leukaemia. Br J Haematol. 2014;167:233–7. 25039477 10.1111/bjh.13035 PMC4188732

[29] Advani AS, Li H, Michaelis LC, Medeiros BC, Liedtke M, List AF, et al. Report of the relapsed/refractory cohort of SWOG S0919: a phase 2 study of idarubicin and cytarabine in combination with pravastatin for acute myelogenous leukemia (AML). Leuk Res. 2018;67:17–20. 29407182 10.1016/j.leukres.2018.01.021 PMC6574123

[30] Boettcher S, Miller PG, Sharma R, Mcconkey M, Leventhal M, Krivtsov AV, et al. A dominant-negative effect drives selection of TP53 missense mutations in myeloid malignancies. Science. 2019;365:599–604. 10.1126/science.aax3649 PMC7327437 31395785

[31] Schavolt KL, Pietenpol JA. p53 and Δnp63α differentially bind and regulate target genes involved in cell cycle arrest, DNA repair and apoptosis. Oncogene. 2007;26:6125–32. 17404570 10.1038/sj.onc.1210441

[32] Tyner JW, Tognon CE, Bottomly D, Wilmot B, Kurtz SE, Savage SL, et al. Functional genomic landscape of acute myeloid leukaemia. Nature. 2018;562:526–31. 30333627 10.1038/s41586-018-0623-z PMC6280667

[33] Giacomelli AO, Yang X, Lintner RE, McFarland JM, Duby M, Kim J, et al. Mutational processes shape the landscape of TP53 mutations in human cancer. Nat Genet. 2018;50:1381–7. 30224644 10.1038/s41588-018-0204-y PMC6168352

[34] Guernet A, Mungamuri SK, Cartier D, Sachidanandam R, Jayaprakash A, Adriouch S, et al. CRISPR-barcoding for intratumor genetic heterogeneity modeling and functional analysis of oncogenic driver mutations. Mol Cell. 2016;63:526–38. 27453044 10.1016/j.molcel.2016.06.017 PMC5537739

[35] Schimmer RR, Kovtonyuk LV, Klemm N, Fullin J, Stolz SM, Mueller J, et al. TP53 mutations confer resistance to hypomethylating agents and BCL-2 inhibition in myeloid neoplasms. Blood Adv. 2022;6:3201–6. 35026842 10.1182/bloodadvances.2021005859 PMC9198927

[36] Ianevski A, Giri AK, Aittokallio T. SynergyFinder 2.0: visual analytics of multi-drug combination synergies. Nucleic Acids Res. 2020;48:488 10.1093/nar/gkaa216 PMC7319457 32246720

[37] Brem EA, O'Brien SM, Becker PS, Jeyakumar D, Pinter-Brown LC, Kirk C, et al. A phase 1 study of adding pitavastatin to venetoclax-based therapy in AML and CLL/SLL. Blood. 2023;142:5940 10.1016/j.bneo.2024.100036 PMC12182841 40552135

[38] DeBose-Boyd RA. Feedback regulation of cholesterol synthesis: Sterol-accelerated ubiquitination and degradation of HMG CoA reductase. Cell Res. 2008;18:609–21. 18504457 10.1038/cr.2008.61 PMC2742364

[39] Lu XY, Shi XJ, Hu A, Wang JQ, Ding Y, Jiang W, et al. Feeding induces cholesterol biosynthesis via the mTORC1–USP20–HMGCR axis. Nature. 2020;588:479–84. 33177714 10.1038/s41586-020-2928-y

[40] Deponte M. Glutathione catalysis and the reaction mechanisms of glutathione-dependent enzymes. Biochim Biophys Acta Gen Subj. 2013;1830:3217–66. 10.1016/j.bbagen.2012.09.018 23036594

[41] Li J, Meng Y, Wu X, Sun Y. Polyamines and related signaling pathways in cancer. Cancer Cell Int. 2020;20:539. 33292222 10.1186/s12935-020-01545-9 PMC7643453

[42] Ha HC, Sirisoma NS, Kuppusamy P, Zweier JL, Woster PM, Casero RA. The natural polyamine spermine functions directly as a free radical scavenger. Proc Natl Acad Sci USA. 1998;95:11140–5. 9736703 10.1073/pnas.95.19.11140 PMC21609

[43] Zahedi K, Barone S, Soleimani M. Polyamines and their metabolism: from the maintenance of physiological homeostasis to the mediation of disease. Med Sci. 2022;10. 10.3390/medsci10030038 PMC9326668 35893120

[44] Falsetti SC, Wang D, Peng H, Carrico D, Cox AD, Der CJ, et al. Geranylgeranyltransferase I inhibitors target RalB to inhibit anchorage-dependent growth and induce apoptosis and RalA to inhibit anchorage-independent growth. Mol Cell Biol. 2007;27:8003–14. 17875936 10.1128/MCB.00057-07 PMC2169159

[45] Kazi A, Carie A, Blaskovich MA, Bucher C, Thai V, Moulder S, et al. Blockade of protein geranylgeranylation inhibits Cdk2-dependent p27 Kip1 phosphorylation on Thr187 and accumulates p27 Kip1 in the nucleus: implications for breast cancer therapy. Mol Cell Biol. 2009;29:2254–63. 19204084 10.1128/MCB.01029-08 PMC2663293

[46] Vogt A, Sun J, Qian Y, Hamilton AD, Sebti M. The geranylgeranyltransferase-I inhibitor GGTI-298 arrests human tumor cells in G0/G1 and induces p21(WAF1/CIP1/SD11) in a p53-independent manner. J Biol Chem. 1997;272:27224–9. 9341167 10.1074/jbc.272.43.27224

[47] Sun J, Qian Y, Chen Z, Marfurt J, Hamilton AD, Sebti M. The geranylgeranyltransferase I inhibitor GGTI-298 induces hypophosphorylation of retinoblastoma and partner switching of cyclin- dependent kinase inhibitors: a potential mechanism for GGTI-298 antitumor activity. J Biol Chem. 1999;274:6930–4. 10066746 10.1074/jbc.274.11.6930

[48] Dan HC, Jiang K, Coppola D, Hamilton A, Nicosia SV, Sebti SM, et al. Phosphatidylinositol-3-OH kinase/AKT and survivin pathways as critical targets for geranylgeranyltransferase I inhibitor-induced apoptosis. Oncogene. 2004;23:706–15. 14737105 10.1038/sj.onc.1207171

[49] Goss GD, Jonker DJ, Laurie SA, Weberpals JI, Oza AM, Spaans JN, et al. A phase I study of high-dose rosuvastatin with standard dose erlotinib in patients with advanced solid malignancies. J Transl Med. 2016;14:83. 27036206 10.1186/s12967-016-0836-6 PMC4815068

[50] Daver NG, Iqbal S, Renard C, Chan RJ, Hasegawa K, Hu H, et al. Treatment outcomes for newly diagnosed, treatment-naïve TP53-mutated acute myeloid leukemia: a systematic review and meta-analysis. J Hematol Oncol. 2023;16:19. 36879351 10.1186/s13045-023-01417-5 PMC9990239

[51] Eriksson M, Ambroise G, Ouchida AT, Lima Queiroz A, Smith D, Gimenez-Cassina A, et al. Effect of mutant p53 proteins on glycolysis and mitochondrial metabolism. Mol Cell Biol. 2017;37. 10.1128/MCB.00328-17 PMC5705820 28993478

[52] Abdullah MI, de Wolf E, Jawad MJ, Richardson A. The poor design of clinical trials of statins in oncology may explain their failure – lessons for drug repurposing. Cancer Treat Rev. 2018;69:84–89. 29936313 10.1016/j.ctrv.2018.06.010

[53] Juarez D, Fruman DA. Targeting the mevalonate pathway in cancer. Trends Cancer. 2021;7:525–40. 33358111 10.1016/j.trecan.2020.11.008 PMC8137523

[54] Schonewille M, Freark de Boer J, Mele L, Wolters H, Bloks VW, Wolters JC, et al. Statins increase hepatic cholesterol synthesis and stimulate fecal cholesterol elimination in mice. J Lipid Res. 2016;57:1455–64. 27313057 10.1194/jlr.M067488 PMC4959861

[55] De Wolf E, Abdullah MI, Jones SM, Menezes K, Moss DM, Drijfhout FP, et al. Dietary geranylgeraniol can limit the activity of pitavastatin as a potential treatment for drug-resistant ovarian cancer. Sci Rep. 2017;7:5410 28710496 10.1038/s41598-017-05595-4 PMC5511264

[56] Juarez D, Buono R, Matulis SM, Gupta VA, Duong M, Yudiono J, et al. Statin-induced mitochondrial priming sensitizes multiple myeloma cells to BCL2 and MCL-1 inhibitors. Cancer Res Commun. 2023;3:2497–509. 37956312 10.1158/2767-9764.CRC-23-0350 PMC10704957

[57] Jiao Z, Cai H, Long Y, Sirka OK, Padmanaban V, Ewald AJ, et al. Statin-induced GGPP depletion blocks macropinocytosis and starves cells with oncogenic defects. Proc Natl Acad Sci USA. 2020;117:4158–68. 32051246 10.1073/pnas.1917938117 PMC7049144

[58] Shu X, Wu J, Zhang T, Ma X, Du Z, Xu J, et al. Statin-induced geranylgeranyl pyrophosphate depletion promotes ferroptosis-related senescence in adipose tissue. Nutrients. 2022;14. 10.3390/nu14204365 PMC9607568 36297049

[59] Chew T, Yannakou CK, Ganju V, Grant S, Sebti S, Prince HM. Phase 1 pharmacodynamic and pharmacokinetic study of the geranylgeranyltransferase I inhibitor PTX-100 (GGTI-2418) in patients with advanced malignancies. Blood. 2023;142:1705

