# Supplementary material for: Chemoresistance of TP53 mutant acute myeloid leukemia requires the mevalonate byproduct, geranylgeranyl pyrophosphate, for induction of an adaptive stress response
Source: Leukemia. 2025 Jul 9;39(9):2087–98. doi: 10.1038/s41375-025-02668-6 (PMC12380605; doi:10.1038/s41375-025-02668-6)

Supplemental Methods:

Reagents – Cytarabine (Hospira) was obtained from The Hospital of The University of Pennsylvania (UPenn) and diluted in H_2O. Rosuvastatin (Sigma), pitavastatin (MedChemExpress), GGTI298 (Sigma), GGTI2417 (Provided by Dr. Said Sebti), and FTI277 (Sigma) were dissolved in DMSO. Reduced glutathione (Sigma) was directly dissolved in media and buffered with NaOH (Sigma). Mevalonolactone (Sigma) is provided in ethanol. Geranylgeranyl pyrophosphate (Sigma) is provided in methanol. Drugs are diluted so that final vehicle concentration is 0.1% weight/volume.

Flow sorting – Patient samples were thawed and washed with PBS supplemented with 5% FBS (Corning) supplemented with DNase I (Sigma) at 50μg/mL. Cells were incubated with a fixable viability stain 510 (FVS510, BD Biosciences) for 15 min at room temperature, washed, and then stained with antibodies against human CD45-BV786 (BD Biosciences #563716), CD33-BV421 (BD Biosciences #565949), CD3-APCR700 (BD Biosciences #565119), and CD19-BB515 (BD Biosciences #564456) for 20 min at room temperature. Cells were then washed and resuspended in PBS supplemented with 2% FBS and flow sorted to isolate viable ($FVS510^-$) $CD3^-$/$CD19^-$/CD45+ / CD33+ cells using a BD FACSAria III cell sorter.

RNA-Sequencing Analysis – Following flow sorting, primary samples were washed in PBS and then extraction was performed using the Qiagen AllPrep DNA/RNA kit. The purity and concentration of the extracted DNA and RNA were assessed using a Nanodrop before downstream applications. RNA was sent to Tempus for library preparation and sequencing on an Illumina HiSeq. RNA libraries were prepared from PolyA selected mRNA species. Multiplexed sequencing was performed with paired-ends to a read depth of 20-30 million per sample. FASTQ files were aligned to hg19 using STAR Align version 2.7.8a[1]. Genes were counted using HTSeq-Count version 2.0.3[2]. Gene expression data in the form of counts per million was log2 transformed with a pseudocount of 0.5 added to all values and z-scored. Single sample gene set enrichment analysis was performed using the GSVA package in R. Gene signatures were tested from the MsigDB C2 or Hallmark collection. Statistical differences between *TP53*^{mut} and *TP53*^{WT} patients were assessed by Student's T-test. RNA sequencing analysis was also performed on *de novo* AML patients from the BeatAML cohort[3] obtained from the public database[3].

Generation of isogenic *TP53*^{mut} MOLM14 AML cell lines – sgRNA sequences for negative control (Rosa) and TP53 targeting (Gia 5 and Grum A) are as follows: GAAGATGGGCGGGAGTCTTC, CATGTGTAACAGTTCCTGCA and GGGCAGCTACGGTTTCCGTC, respectively[4,5]. These were ligated into LentiCRISPRv2-mCherry (Addgene #99154), digested with BsmBI (NEB #R0580). Lentivirus was packaged in 293T cells cotransfected with pHEF-VSVG (Addgene#22501) and pPax2 (Addgene#12259) in PEI transfection reagent. Lentivirus supernatant was harvested at 2- and 3-days post transfection and was concentrated with PegIT Precipitation (SBI #LV810A-1). MOLM14 cells were then incubated with virus overnight and transduction was verified by flow cytometry for mCherry. Mutations were validated by DNA sequencing and loss of p53 function confirmed by Western blotting after AraC, as described above. Single cell clones were selected by serial dilution and expanded. p53 loss of function was re-confirmed in all single cell clones.

DNA sequencing – Cell lines were washed twice with PBS and flash frozen as pellets. Subsequently, genomic DNA was isolated using a the Qiagen DNeasy Blood and Tissue Kit. DNA from primary samples was extracted with the Qiagen AllPrep DNA/RNA kit as described above. Libraries of germline DNA were prepared using KAPA Hyper Prep Kit (Roche Diagnostics, Branchburg NJ). Libraries were subjected to targeted next generation sequencing (NGS) of the *TP53* gene on an Illumina Nova-Seq. FASTQ files from sequencing were aligned to human genome version 38 (hg38) using BWA-mem version 0.7.17[6]. Germline variants were called from BAM files using Genome Analysis Toolkits (GATK) HaplotypeCaller version 3.7. The presence of *TP53* variants was validated manually using the Integrative Genomics Viewer version 2.5.2.

Apoptosis assay – Unfixed cells were resuspended in Annexin Binding Buffer (10mM HEPES, 140mM NaCl, 2.5mM $CaCl_2$ pH 7.4) and stained with Annexin V-APC (Thermo Fisher #A35110) and 7AAD (BD Biosciences #559925). Stained cells were analyzed using a BD Accuri C6 flow cytometer. Data was processed with FlowJo version 10 (BD Biosciences).

Immunoblot – Cells were lysed with the CellLytic MT Cell Lysis Reagent (Sigma) with protease and phosphatase inhibitors (Halt Protease & Phosphatase Inhibitor Cocktail; Thermo Fisher). Lysates were analyzed by SDS-PAGE. Immunoblot detection was performed using Amersham ECL Detection Reagents (Cytiva). Antibodies used are as follows: p53 (EMD Millipore #OP43), p21 (BD Pharmingen #556430), HMGCR (Invitrogen #MA5-35242), SREBP2 (Invitrogen #PA1-338), TBP (CST, #44059). Additional reagents used include a SREBP2 positive control lysate (Origene #LC417881) and secondary antibodies (CST #7074, CST #7076). Signal intensity was quantified using ImageJ.

XTT Cell viability assay – Cells were pre-treated with rosuvastatin or DMSO for 24 hours. Cells were then plated in 96-well plates at 100,000 per well in triplicate with AraC plus fresh DMSO or rosuvastatin. Cell viability was measured after 24 hours of AraC treatment using the XTT Cell Viability Kit from Cell Signaling Technology (#9095) and a Tecan Infinite 200 Pro plate reader. Drug-drug interaction was evaluated using the SynergyFinder web-based tool, with a score greater than 10 being likely synergistic, -10 to 10 likely additive, and less than -10 likely antagonistic[7].

Quantitative reverse transcription polymerase chain reaction (qRT-PCR) – AML cell lines were harvested, washed with PBS, and flash frozen in liquid nitrogen as cell pellets. RNA extraction was performed using the QIAGEN RNeasy Mini Kit following the manufacturer's protocol with the following adjustments. Cells were lysed using Buffer RLT supplemented with DNase I to remove genomic DNA and homogenized using QIAshredders. The quantity and quality of isolated RNA was assessed with the Nanodrop One. Total RNA extracted was then reverse transcribed into complementary DNA (cDNA) utilizing the BIO-RAD iScript RT Supermix according to the manufacturer's instructions. qRT-PCR was performed using TaqMan Fast Advanced Master Mix and predesigned TaqMan probes on a 384-well plate using the ViiA 7 Rea-time PCR system (Thermo Fisher). The predesigned TaqMan probes used are as follows: *ABCA1* (Hs00194045), *SREBF1* (Hs02561944), *SREBF2* (Hs01081784), *HMGCR* (Hs00168352), *IDI1* (Hs00743568), *TRIT1* (Hs01091215), *FDPS* (Hs01578769), *GGPS1* (Hs01546492), *FNTA* (Hs00357739), *PGGT1B* (Hs00270701), *RABGGTA* (Hs01554344), *RABGGTB* (HS00190183), *PDSS1* (Hs00372008), *PDSS2*

(Hs01047689), *FDFT1* (Hs00926054), *SQLE* (Hs01123768), *LSS* (Hs01552331), *GAPDH* (Hs02786624), *CDKN1A* (Hs00355782), *MYC* (Hs00153408).

Targeted quantification by liquid chromatography-high resolution mass spectrometry – Cells were resuspended in fresh, pre-warmed media at 400,000 cells per mL and treated as described, with 5 biological replicates per condition. At the end of treatment, cells were counted and 2 million cells per condition were washed twice in cold PBS, flash frozen as cell pellets, and stored at -80 °C until processing at UPenn. The cell pellets were then spiked with a panel of stable isotope labeled lipids as internal standards for normalization of extraction and analysis. The lipid extraction and MS analysis was done as previously described[8].

Metabolomics by ultra high performance liquid chromatography by mass spectrometry – Cells were resuspended in fresh, pre-warmed media at 400,000 cells per mL and treated as described, with 5 biological replicates per condition. At the end of treatment, cells were counted and 2 million cells per condition were washed twice in cold PBS, flash frozen as cell pellets, and shipped on dry ice to the University of Colorado. 1mL of cold 5:3:2 MeOH:ACN:H_2O was added to frozen cell pellets and prepared for a 10μL injection in a high-throughput 5 minute gradient as previously described[9].

Seahorse Assay – All XF assays were performed using the Agilent Seahorse XFe96 Extracellular Flux Analyzer. The day before the assay, the sensor cartridge was placed into the calibration buffer medium supplied by Seahorse Biosciences to hydrate overnight. Seahorse XFe96 microplates wells were coated with 25μL of Cell-Tak (Corning; Cat#354240) solution at a concentration of 22.4μg/ml at room temperature for 20 minutes, washed twice with distilled water, and kept at 4 °C overnight. On the day of the experiment, AML cells were plated at a density of 80,000 cells per well for cell lines and 100,000 cells per well for primary samples in XF base minimal DMEM media containing 11mM glucose, 1mM pyruvate and 2mM glutamine. Then 180μL of XF base minimal DMEM medium was added to each well and the microplate was centrifuged at 100 g for 1 min with no break. After no more than one hour of incubation at 37 °C in CO_2 free-atmosphere, basal oxygen consumption rate (OCR, as a mitochondrial respiration indicator) and extracellular acidification

rate (ECAR, as a glycolysis indicator) were performed using the Seahorse XF Cell Mito Stress Test Kit (#103015-100).

Mitochondrial DNA quantification – Genomic DNA was extracted using the Qiagen DNeasy Blood and Tissue Kit. The purity and concentration of the extracted DNA was assessed using a Nanodrop before downstream applications. PCR was performed using the PowerTrack SYBR Green Master Mix and the primers listed below to determine the ratio of mitochondrial to nuclear DNA using a Thermo Fisher Scientific ViiA 7 Real-Time PCR System. Mitochondria DNA (*ND2)*: forward primer sequence – CCTATCACCCTTGCCATCAT; reverse primer sequence – GAGGCTGTTGCTTGTGTGAC. Nuclear DNA (*Pecam1*): forward primer sequence – ATGGAAAGCCTGCCATCATG; reverse primer sequence – TCCTTGTTGTTCAGCATCAC.

Measurement of ROS content and mitochondrial mass – Mitochondrial ROS content and mass in viable cells were measured by flow cytometry using MitoSOX Red Mitochondrial Superoxide Indicator (Thermo Fisher #M36007) and Tom20 Antibody (5-10) Alexa Fluor488 (Santa Cruz #17764).

Glutathione Assay – Total glutathione and the ratio of reduced to oxidized glutathione (GSH/GSSG) were determined using the GSH-Glo Glutathione Assay from Promega, which is a luminescence-based assay. Assay was performed and analyzed by their protocol using 25,000 cells per well in triplicate and analyzed with a Tecan Infinite 200 Pro plate reader.

Colony-forming assays – Primary human AML cells were plated in cytokine-enriched methylcellulose (R&D Systems HSC005) in triplicate to allow for statistical analysis and based on the number of human AML cells available. Plates were maintained at 37 °C and 5% CO_2. Drug treatments were added only at the time of plating. Human mononuclear cells were seeded at 10,000-150,000 cells per plate for AML. Colonies were scored at 14 days. Only data from samples with growth of >30 colonies per plate in the DMSO control condition were included.

Patient-derived xenograft model – Animals were used in accordance with a protocol reviewed and approved by the Institutional Animal Care and Use Committee at UPenn. NOD/LtSz-SCID/IL-2Rγchain^{null} (NSG) mice were produced at The Stem Cell and Xenograft Core using breeders obtained from The Jackson Laboratory. Mice were housed and human primary AML cells were transplanted as reported previously[10]. Briefly, mice were housed in sterile conditions using HEPA-filtered microisolators and fed with irradiated food and sterile water. Mice (6–9 weeks old) were sublethally treated with busulfan (30mg/kg/day) 24 hours before injection of leukemic cells. Leukemia samples were thawed at room temperature, washed twice in PBS, and suspended in PBS at a final concentration of 10-20 million cells per mL. 100 μL (1-2 million cells) was injected into the tail vein of each mouse. Daily monitoring of mice for symptoms of disease (ruffled coat, hunched back, weakness, and reduced mobility) determined the time of killing for injected animals with signs of distress. If no signs of distress were seen, mice were initially analyzed for engraftment 8 weeks after injection except where otherwise noted.

In vivo drug administration – Four to 12 weeks after AML cell transplantation and when mice were engrafted (confirmed by flow cytometry on bone marrow aspirates), 7 animals were assigned to one of four cohorts by a random number generator with approximately equal average engraftment and body weight across the cohorts. Seven mice per primary group was chosen to achieve 99% power with an alpha of 0.05 using two-sample t-tests with unequal variance based on prior experimental data. The mice were treated by daily intraperitoneal injection of AraC (Hospira) 50mg/kg for 5 days or Vehicle (PBS) or daily oral administration of rosuvastatin (Sigma) 1mg/kg for 7 days or Vehicle (4% DMSO, 30% PEG-300, double distilled H_2O). Mice were monitored for toxicity and provided nutritional supplements as needed. Investigators were unblinded during treatment of mice, but blinded during final processing by only utilizing mouse tag number rather than treatment cohort.

Assessment of leukemic engraftment – Mice were harvested on days 8 and 9 following initiation of drug treatment. NSG mice were humanely killed in accordance with ethics protocols. Bone marrow (mixed from tibias and femurs) and spleen were dissected in a sterile environment and crushed in 15mL PBS with 2% FBS and filtered through 40μM filters. Samples were centrifuged and resuspended in 2mL PBS with 2%

FBS. 100μL per sample was removed for assessment of leukemic engraftment by flow cytometry. 50uL antibody mix was added and cells were incubated for 20 minutes in the dark at room temperature. Antibody mix was composed of human CD45-PE (BD Biosciences #555483), mouse CD45-PE-Cy7 (BD Biosciences #552848), CD33-BB515 (BD Biosciences #564588), CD3-BV421 (BD Biosciences #562426) and CD19-BB700 (BD Biosciences #566396). 750μL of 1X BD FACS Lysing Solution was added to each tube and incubated for 15 minutes at room temperature. Cells were washed and resuspended in 300μL of 2% paraformaldehyde. 20μL of CountBright Absolute Counting Beads (Invitrogen) were added and immediately vortexed. Analyses were performed on a Life Science Research II (LSR II) flow cytometer with DIVA software (BD Biosciences). The number of AML cells/μL bone marrow or spleen were determined by using the CountBright beads protocol (Invitrogen).

<u>Magnetic bead separation and downstream processing of leukemic cells in the PDX model</u> – Following 100μL removal for flow cytometry, 1.9mL of remaining BM sample was centrifuged and then lysed with ammonium chloride for 5 minutes. Cells were washed, centrifuged and then processed for magnetic bead separation using human CD45 microbeads (Miltenyi) *via* their protocol using the Miltenyi autoMACS. Following cell harvest, cells were resuspended in PBS with 2% FBS and divided for flow cytometry sort purity determination, MS or Seahorse. For MS, 3-5 replicates of 0.5 million cells per mouse were washed and then flash frozen as cell pellets and stored at -80 °C. For Seahorse, cells were resuspended at 1 million cells per mL in IMDM supplemented with 20% FBS. After 1 hour incubation at 37°C and 5% CO_2 cells were processed for Seahorse analysis per above.

<u>Retrospective clinical data</u> – 364 total patients diagnosed with *TP53*^{mut} AML were evaluated, including 215 treated at UPenn and 149 from Roswell Park Comprehensive Cancer Center (Roswell). At UPenn, patients can enroll and provide consent for an IRB-approved (#703185) study overseen by the UPenn SCXC for the collection of data from the electronic health record. At Roswell, patients are consented for use of their electronic medical record in a deidentified fashion at the time of their initial visit (IRB Protocol #BDR173423). We identified AML patients with a *TP53* mutation at the time of diagnosis between 2013 and 2023. We

performed chart and/or database reviews to determine treatment with a statin before and/or during initial treatment for AML as well as baseline patient demographics and clinical outcomes. Kaplan-Meier methodology was performed for median overall survival.

Supplemental References:

1 Dobin A, Davis CA, Schlesinger F, Drenkow J, Zaleski C, Jha S *et al.* STAR: Ultrafast universal RNA-seq aligner. *Bioinformatics* 2013; **29**. doi:10.1093/bioinformatics/bts635.

2 Anders S, Pyl PT, Huber W. HTSeq-A Python framework to work with high-throughput sequencing data. *Bioinformatics* 2015; **31**. doi:10.1093/bioinformatics/btu638.

3 Tyner JW, Tognon CE, Bottomly D, Wilmot B, Kurtz SE, Savage SL *et al.* Functional genomic landscape of acute myeloid leukaemia. *Nature* 2018; **562**. doi:10.1038/s41586-018-0623-z.

4 Giacomelli AO, Yang X, Lintner RE, McFarland JM, Duby M, Kim J *et al.* Mutational processes shape the landscape of TP53 mutations in human cancer. Nat Genet. 2018; **50**. doi:10.1038/s41588-018-0204-y.

5 Guernet A, Mungamuri SK, Cartier D, Sachidanandam R, Jayaprakash A, Adriouch S *et al.* CRISPR-Barcoding for Intratumor Genetic Heterogeneity Modeling and Functional Analysis of Oncogenic Driver Mutations. *Mol Cell* 2016; **63**. doi:10.1016/j.molcel.2016.06.017.

6 Li H, Durbin R. Fast and accurate short read alignment with Burrows-Wheeler transform. *Bioinformatics* 2009; **25**. doi:10.1093/bioinformatics/btp324.

7 Ianevski A, Giri AK, Aittokallio T. SynergyFinder 2.0: Visual analytics of multi-drug combination synergies. *Nucleic Acids Res* 2021; **48**. doi:10.1093/NAR/GKAA216.

8 Wang D, Ho ES, Cotticelli MG, Xu P, Napierala JS, Hauser LA *et al.* Skin fibroblast metabolomic profiling reveals that lipid dysfunction predicts the severity of Friedreich's ataxia. *J Lipid Res* 2022; **63**. doi:10.1016/j.jlr.2022.100255.

9 Nemkov T, Hansen KC, D'Alessandro A. A three-minute method for high-throughput quantitative metabolomics and quantitative tracing experiments of central carbon and nitrogen pathways. *Rapid Communications in Mass Spectrometry* 2017; **31**. doi:10.1002/rcm.7834.

10 Farge T, Saland E, de Toni F, Aroua N, Hosseini M, Perry R *et al.* Chemotherapy-resistant human acute myeloid leukemia cells are not enriched for leukemic stem cells but require oxidative metabolism. *Cancer Discov* 2017; **7**. doi:10.1158/2159-8290.CD-16-0441.

Supplemental Figure Legends:

Supplemental Figure 1: (A) Principle component analysis from Figure 1A with labelling of each patient sample. For B-D, RNA sequencing from a cohort of the BeatAML dataset comparing *de novo*, untreated primary *TP53*^{mut} (n=17) *versus TP53*^{WT} (n=85) AML samples was analyzed for (B) GSEA scores from the Hallmark and other denoted gene sets, and (C) single sample GSEA with calculation of normalized signature scores for “TP53 Targets,” “Hallmark Cholesterol,” and “Maxwell Cholesterol.” Statistical analysis by Student’s T Test. p-values: * = <0.05, ** = <0.01, *** = <0.001. (D) Kaplan-Meier survival estimates for the primary RNA sequencing BeatAML cohort with statistical analysis by long-rank test. Median overall survival was 165 days for *TP53*^{mut} AML patients and 520 days for the *TP53*^{WT} cohort (log-rank p < 0.0001).

Supplemental Figure 2: (A) Protein expression of p53 and p21 normalized to TBP in representative isogenic *TP53*^{mut} and *TP53*^{WT} MOLM14 AML clones treated for 0, 6 or 24 hours with AraC (1μM) performed by western blot. (B) Protein expression of p53 and p21 normalized to TBP in the four *TP53*^{WT} MOLM14 AML clones collected 6 hours after irradiation (10 Gy) or no irradiation and evaluated by western blot. (C) Cell viability of isogenic *TP53*^{WT} MOLM14 AML clones treated for 24 hours with rosuvastatin (50 μM) followed by an additional 24 hours of vehicle or AraC (1μM) and assessed by flow cytometry following staining with AnnexinV and 7AAD (n=5, 4, 6, respectively). (D) Cell viability of representative isogenic MOLM13 *TP53*^{WT} (M13-WT), MOLM13 *TP53* complete knockout (M13-KO), and MOLM13 *TP53* R273H hotspot missense mutant (M13-R273H) AML clones pretreated with 24 hours of rosuvastatin (50μM) followed by an additional 24 hours of AraC (1μM) and assessed by flow cytometry following staining with AnnexinV and 7AAD (n=4). (E) Cell viability of *TP53* mutant HL60 AML cells treated with vehicle or rosuvastatin (75μM) followed by an additional 24 hours of increasing doses of AraC (0, 1, 2, 4, 8 μM) and assessed by flow cytometry following staining with AnnexinV and 7AAD (n=3) with a chart summarizing the AraC-Rosuvastatin drug-drug

interaction by two-way ANOVA. (F) Dose response matrix of M14-WT1 (left) and M14-Mut1 (right) pretreated with 24h of rosuvastatin (0, 25, 50 or 100μM) followed by an additional 24h of AraC (0, 0.5, 1, 2, 4, 8, 16 μM) and assessed by the XTT assay and the Bliss mean synergy score calculator with a score greater than 10 indicating synergy (n=3). M14-Mut1 mean bliss score 30 and M14-WT1 mean bliss score 36. (G) Cell viability of isogenic *TP53*^{mut} and *TP53*^{WT} MOLM14 AML clones treated for 24 hours with pitavastatin (PIT; 0, 0.1, 0.3, 1, 3, 10 μM) followed by an additional 24 hours of vehicle or AraC (1μM) and assessed by flow cytometry following staining with AnnexinV and 7AAD. Statistical analysis by multiple T Tests with FDR correction for multiple comparisons with q values listed in table below figures. (H) Gene expression of mevalonate pathway genes in isogenic *TP53*^{mut} and *TP53*^{WT} MOLM14 AML clones at 0, 6, 12, 18, and 24-hours with AraC (1μM) with all AraC conditions compared to the 0 hour timepoint for each clone after normalization to GAPDH and as measured by qRT-PCR (n=3). (I) ImageJ quantification presented as relative units (R.U.) of HMGCR protein expression normalized to TBP (n=4).

Supplemental Figure 3: (A) Oxygen consumption in pmol per minute in M14-Mut2 and M14-Mut4 treated with vehicle or AraC (1μM) for 24 hours as measured by Seahorse technology. AraC *versus* vehicle fold-changes: Basal OXPHOS of 1.9 ($p < 0.001$) for M14-Mut2 and 1.9 ($p = 0.03$) for M14-Mut4; Maximum-uncoupler induced OXPHOS of 2 ($p = 0.03$) for M14-Mut2 and 2.2 ($p = 0.04$) for M14-Mut4. Summary of (C) basal and (D) maximum uncoupler-induced oxygen consumption in pmol per minute measured by Seahorse technology in the isogenic MOLM14 *TP53*^{mut} and M14-WT1 AML clones treated with vehicle or AraC (1μM) for 24 hours. (E-G) M14-WT2, M14-WT3, and M14-WT4 were treated with vehicle or AraC (1μM) for 24 hours and oxygen consumption in pmol per minute was measured by Seahorse technology and presented as (E) representative seahorse tracings of the oxygen consumption rate in pmol per minute, (F) summary of basal oxygen consumption rate in pmol per minute (n=4), and (G) summary of maximum uncoupler-induced oxygen consumption in pmol per minute (n=4). AraC *versus* vehicle fold-changes: Basal OXPHOS of 1.0 ($p = 0.16$) for M14-WT2, 0.6 ($p < 0.001$) for M14-WT3, 0.3 ($p = .001$) for M14-WT4; Maximum-uncoupler induced OXPHOS of 1.0 ($p = 0.7$) for M14-WT2, 0.8 ($p = 0.005$) for M14-WT3, 0.8 ($p = 0.4$) for M14-WT4. (H-J) *TP53* mutant HL60 cells were treated with vehicle or AraC (1μM) for 24 hours and oxygen

consumption in pmol per minute was measured by Seahorse technology and presented as (H) representative seahorse tracings of the oxygen consumption rate in pmol per minute, (I) summary of basal oxygen consumption rate in pmol per minute (n=4), and (J) summary of maximum uncoupler-induced oxygen consumption in pmol per minute (n=4). AraC *versus* vehicle fold-changes: Basal OXPHOS of 1.5 (p = .003) for HL60; Maximum-uncoupler induced OXPHOS of 1.9 (p = .002) for HL60. (K) Metabolites in M14-WT1, M14-Mut1, and M14-Mut3 AML cell lines treated with vehicle, 18h AraC (1μM), or 24h AraC (1μM) (n=5 per condition) presented as the ratio to respective cell line vehicle. Statistical analysis by Student's T Test with FDR correction for multiple comparisons for C-D, F-G, and I-J and Z-tests with Bonferroni's multiple comparisons test for L. Adjusted p-values: * = <0.05, ** = <0.01, *** = <0.001. n is the number of replicates.

Supplemental Figure 4: (A-I) Specified cells were treated for a total of 48 hours with rosuvastatin (50μM except 75μM for HL60) with AraC (1μM) added for the last 24 hours, and the following experiments were performed: (A) Maximum uncoupler-induced oxygen consumption rate in pmol per minute assessed by Seahorse technology in M14-WT1, M14-Mut1, and M14-Mut4 (n=7), (B) representative seahorse tracings for M14-WT1 (top), M14-Mut1 (middle), and M14-Mut4 (bottom), (C) basal oxygen consumption rate and (D) maximum uncoupler-induced oxygen consumption rate in pmol per minute assessed by Seahorse technology in M14-WT2, M14-WT3, and M14-WT4 (n=8), (E) Basal oxygen consumption rate and (F) maximum uncoupler-induced oxygen consumption rate in pmol per minute assessed by Seahorse technology in HL60 (n=8), (G-H) representative seahorse tracings for (G) M14-WT2 (top), M14-WT3 (middle), and M14-WT4 (bottom) and (H) HL60, and (I) the ratio of reduced to oxidized glutathione (GSH/GSSG) assessed in 25,000 cells per replicate (n=4, n=5, n=3 for respective cell lines). Statistical analysis by two-way ANOVA with FDR correction for multiple comparisons. p-values: * = <0.05, ** = <0.01, *** = <0.001. n is the number of replicates.

Supplemental Figure 5: All experiments in Supplemental Figures 5A-E were performed in the M14-Mut1 clone pretreated for 24 hours with rosuvastatin (50μM) and either vehicle, MVA (200μM) or GGPP (1μM) followed by an additional 24 hours with vehicle or AraC (1μM) and the subsequent assessment of (A) cell viability presented as the percentage of annexinV and 7AAD negative cells by flow cytometry with or without

MVA (n=3), (B) basal oxygen consumption rate in pmol/minute as assessed by Seahorse technology with or without MVA (n=8), (C) maximum uncoupler-induced oxygen consumption rate in pmol/minute as assessed by Seahorse technology with or without MVA (n=8), (D) representative seahorse tracings presented as oxygen consumption rate (pmol/min) for Fig 5C and Supplemental Fig 5A, (E) maximum uncoupler-induced oxygen consumption rate in pmol/minute as assessed by Seahorse technology with or without GGPP (n=8), (F) representative seahorse tracings presented as oxygen consumption rate (pmol/min) for Fig 5E and Supplemental Fig 5C, and (G) ratio of reduced to oxidized glutathione (GSH/GSSG) assessed in 25,000 cells treated with or without GGPP (n=5). (H) CoQ10 (ng) per 2 million cells were assessed by LC-HRMS in either M14-WT1 or M14-Mut1 cells pretreated for 24 hours with vehicle or rosuvastatin (50μM) and either vehicle or GGPP (1μM) followed by an additional 24 hours with vehicle or AraC (1μM) (n=5). (I) Cell viability as percentage of annexinV and 7AAD negative cells by flow cytometry of M14-Mut4 cells pretreated for 24 hours with vehicle or rosuvastatin (50μM) and either vehicle or GGTI2417 (4μM) followed by an additional 24 hours with vehicle or AraC (1μM) (n=4). The doublet of rosuvastatin and GGTI2417 and the triplicate with AraC were both synergistic [$F(1,24)=23.3$, $p < 0.001$); $(F,1.24)=5.1$, $p = 0.03$), respectively]. (J-K) Cell viability as percentage of annexinV and 7AAD negative cells by flow cytometry of M14-Mut1 cells pretreated for 24 hours with vehicle or rosuvastatin (50μM) and either vehicle or (J) GGTI298 (10μM; n=5) or (K) FTI277 (10μM; n=4) followed by an additional 24 hours with vehicle or AraC (1μM). Statistical analysis by two-way ANOVA with FDR correction for multiple comparisons. Adjusted p-values: * = <0.05, ** = <0.01, *** = <0.001. n is the number of replicates.

Supplemental Figure 6: All experiments were performed in the M14-WT1 clone pretreated for 24 hours with vehicle or rosuvastatin (50μM) and either vehicle, MVA (200μM), GGPP (1μM), GGTI417 (4 μM), GGTI298 (10μM), or FTI277 (10μM) (as indicated in figure) followed by an additional 24 hours with vehicle or AraC (1μM) with the subsequent assessment of (A) cell viability as the percentage of annexinV and 7AAD negative cells by flow cytometry with or without MVA (n=3), (B) basal oxygen consumption rate in pmol/minute as assessed by Seahorse technology with or without MVA (n=8), (C) maximum uncoupler-induced oxygen consumption rate in pmol/minute as assessed by Seahorse technology with or without MVA

(n=8), (D) representative Seahorse tracings presented as oxygen consumption rate (pmol/min) for Supplemental Fig 6B-C, (E) cell viability as the percentage of annexinV and 7AAD negative cells by flow cytometry with or without GGPP (n=5), (F) basal oxygen consumption rate in pmol/minute as assessed by Seahorse technology with or without GGPP (n=8), (G) maximum uncoupler-induced oxygen consumption rate in pmol/minute as assessed by Seahorse technology with or without GGPP (n=8), (H) representative Seahorse tracings presented as oxygen consumption rate (pmol/min) for Supplemental Fig 6F-G, (I) TOMM20 presented as MFI in viable cells (by FVS-R) with or without GGPP (n=4), (J) mitochondrial ROS presented as the MFI of MitoSox in viable cells (by FVS-R) with or without GGPP (n=3), (K) total glutathione presented as μM per 25,000 cells with or without GGPP (n=4), (L) ratio of reduced to oxidized glutathione (GSH/GSSG) assessed in 25,000 cells treated with or without GGPP (n=4), and cell viability as the percentage of annexinV and 7AAD negative cells by flow cytometry with or without (M) GGTI2417 (n=3), (N) GGTI298 (n=4) or (O) FTI277 (n=3). Statistical analysis by two-way ANOVA with FDR correction for multiple comparisons. Adjusted p-values: * = <0.05, ** = <0.01, *** = <0.001. n is the number of replicates.

Supplemental Figure 7: (A) TOMM20 presented as MFI in viable cells (by FVS-R) assessed by flow cytometry in previously frozen viable samples (SCXC-4708, -6865, -5052, -7575) that were resuspended in X-Vivo media with 20% BIT serum and 10ng/mL of cytokines FLT3, SCF, IL3 and IL6, pretreated with rosuvastatin (50μM) for 24 hours followed by AraC (1μM) treatment for an additional 24 hours with 1 replicate per condition. (B) Oxygen consumption rate (pmol/min) assessed by Seahorse technology in cells that were conditioned as per (A) followed by dead cell depletion *via* the AnnexinV dead cell removal kit and plating for Seahorse analysis. 5 technical replicates per condition. AraC *versus* vehicle fold-changes were as follows: 1.1-fold (p = 0.13) and 1.1-fold (p = 0.2) for basal and max OXPHOS, respectively. (C) Total number of colony forming units assessed 14 days after plating previously viably frozen primary *TP53*^{mut} AML samples (SCXC-7575) treated on day 0 with rosuvastatin (15μM or 30μM) and/or AraC (5nM or 10nM) with 3 replicates per condition. Statistical analysis by one-way ANOVA with FDR correction for multiple comparisons. q values provided in the figure. n is the number of replicates.

Supplemental Figure 8: (A) Oxygen consumption rate tracings in pmol/minute for Fig 7C. Top panel includes all seahorse tracings for each PDX experiment, with each line representing 1 mouse. Subsequent panels focus specifically on vehicle *versus* AraC, Statin, and AraC with Rosuvastatin, respectively. (B) Leukemic burden in millions of hCD45+ hCD33+ cells (chemosensitive sample SCXC-7575) in the bone marrow and spleen combined for each mouse and quantified with counting beads by flow cytometry. (C) Basal and maximum coupler-induced oxygen consumption in pmol/minute assessed by Seahorse technology in magnetic bead-purified hCD45+ leukemic cells from the bone marrow with each circle representing the average of 1 mouse (n=4, 3, 2, 2 mice for Control, AraC, Statin and AraC + Statin, respectively; chemosensitive sample SCXC-7575). (D) Oxygen consumption rate tracings in pmol/minute for Supplemental Fig 8C.

Supplemental Table 1: (A) Clinical and demographic characteristics of primary AML samples included in the RNA sequencing experiment. Summary of (B) differential gene expression and (C) GSEA in primary *TP53*^{mut} (n=9) *versus* *TP53*^{WT} (n=21) AML patient samples. (D) List of additional gene sets evaluated in the GSEA. (E) Characteristics of cell lines and patient samples utilized in Figures 2-7, including focused *TP53* sequencing of the isogenic *TP53*^{mut} and *TP53*^{WT} MOLM14 AML cell line clones. (F) Metabolites as obtained by MS in M14-WT1, M14-Mut1, and M14-Mut3 AML cell lines treated with vehicle, 18h AraC (1μM), or 24h AraC (1μM) presented as fold-change and associated t-test for the following comparisons: M14-WT1 vehicle *vs* M14-Mut1 or M14-Mut3, 18h AraC (1μM) *vs* vehicle in M14-WT1, M14-Mut1, and M14-Mut3, and 24h AraC (1μM) *vs* vehicle in M14-WT1, M14-Mut1, and M14-Mut3, and 24h AraC (1μM). CmpdID = Compound ID. (G) Clinical and demographic characteristics of the 364 patients evaluated in the retrospective chart review.

Supplemental Figure 1:

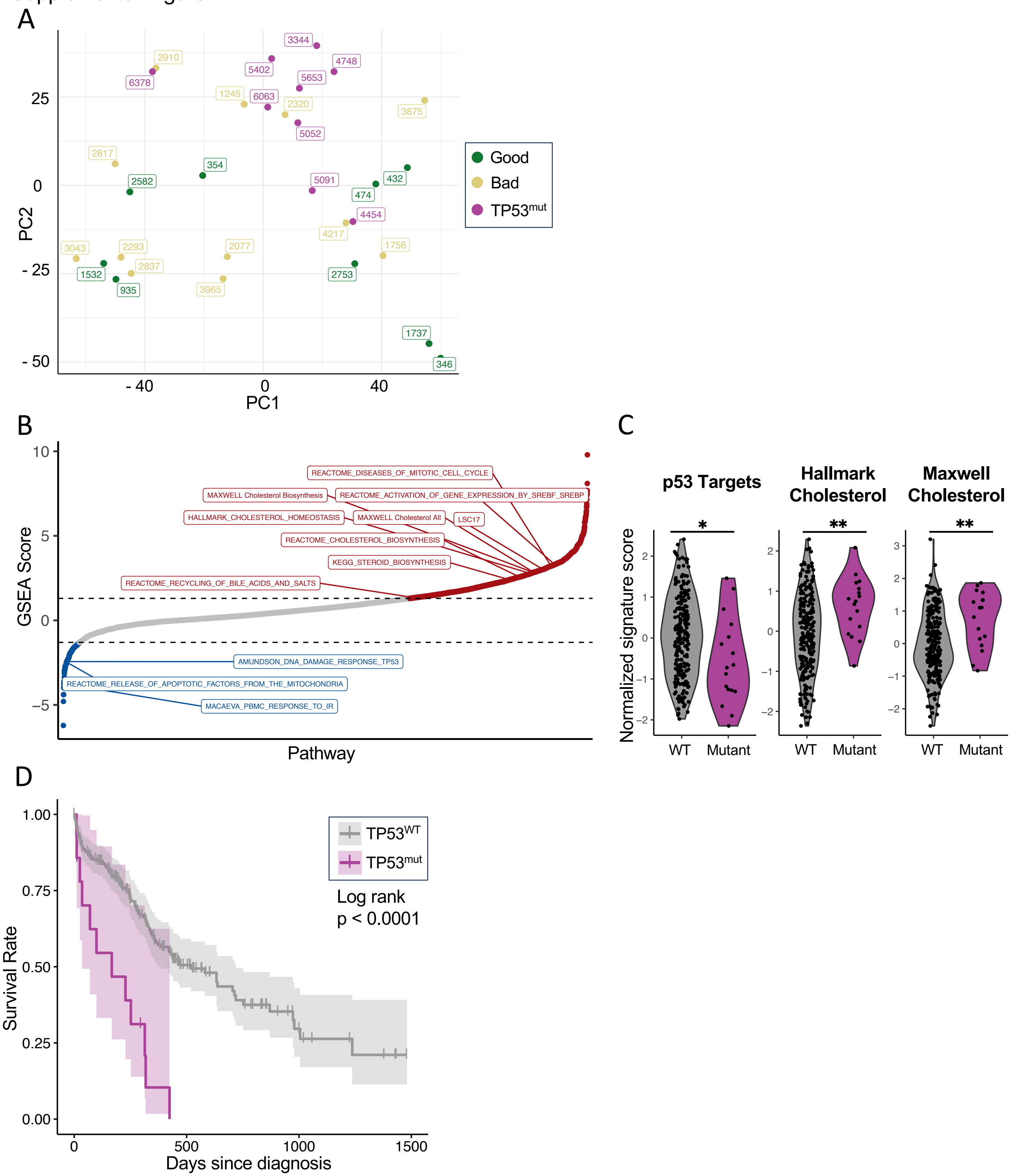

Supplemental Figure 2:

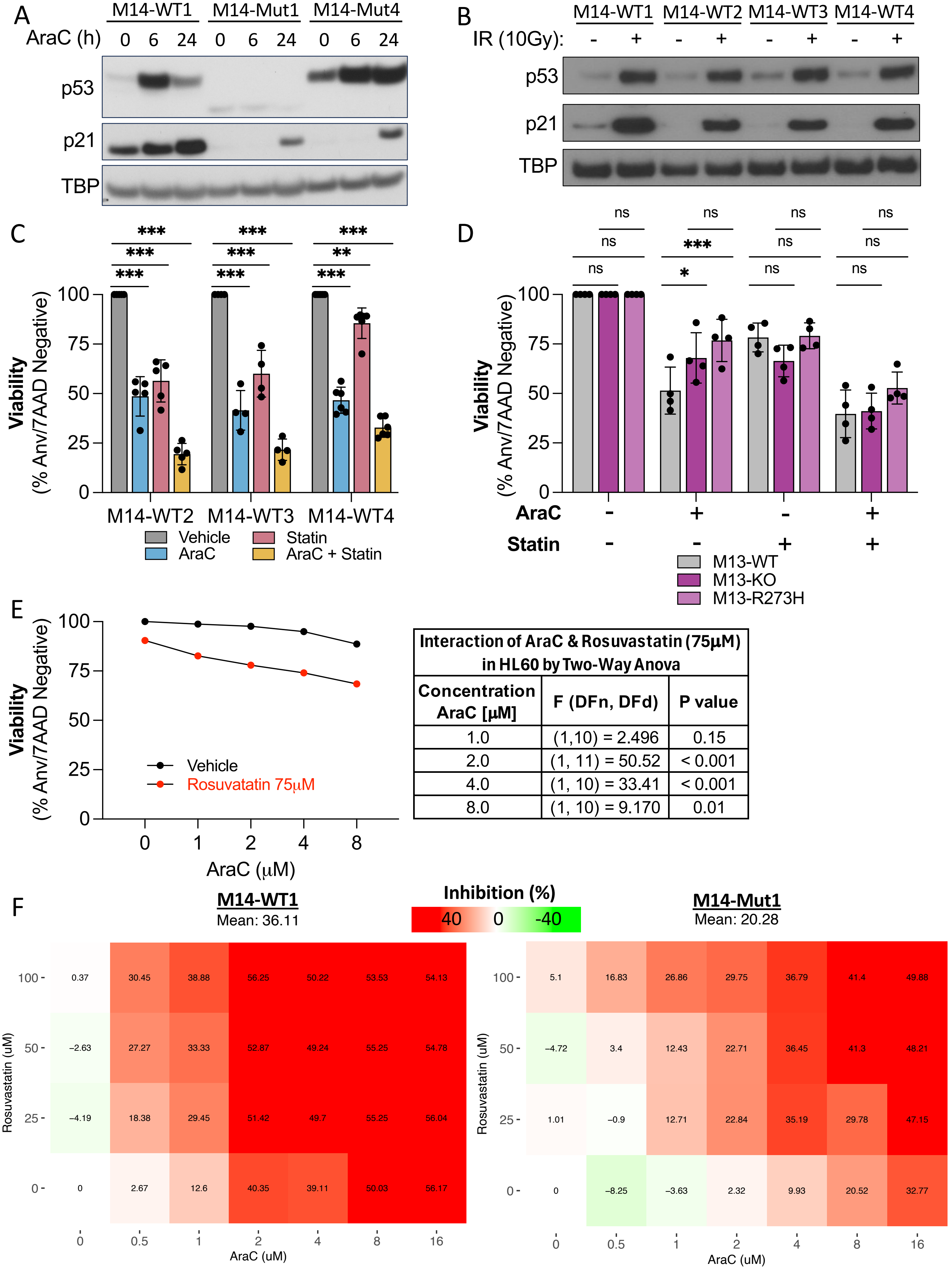

Interaction of AraC & Rosuvastatin (75μM) in HL60 by Two-Way Anova		
Concentration AraC [μM]	F (DFn, DFd)	P value
1.0	(1,10) = 2.496	0.15
2.0	(1, 11) = 50.52	< 0.001
4.0	(1, 10) = 33.41	< 0.001
8.0	(1, 10) = 9.170	0.01

Supplemental Figure 2 Continued:

G

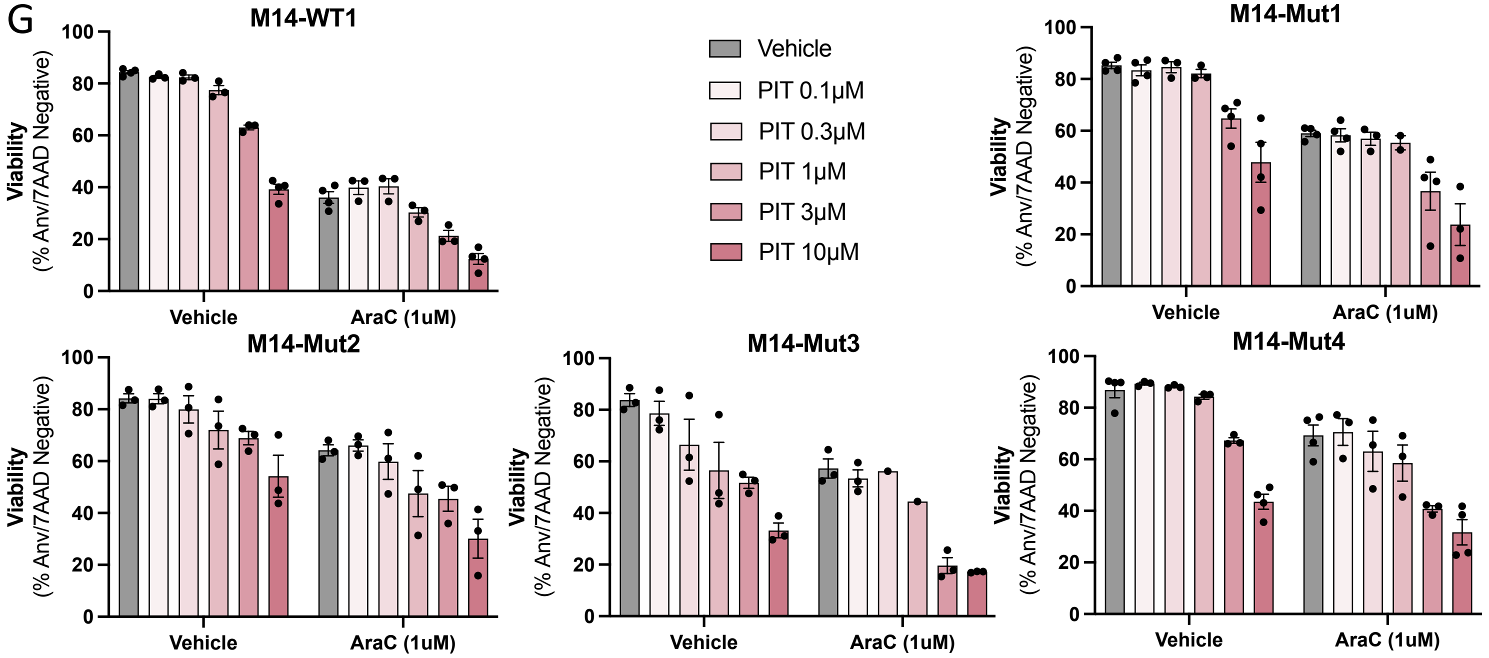

q-values<br>Comparing viability of pitavastatin-treated cells with or without AraC co-treatment					
Pitavastatin Concentration [uM]	M14-WT1	M14-Mut1	M14-Mut2	M14-Mut3	M14-Mut4
0.0	0.000005	0.00001	0.010573	0.008517	0.029681
0.1	0.000107	0.000258	0.010573	0.014274	0.029681
0.3	0.000163	0.000767	0.100977	0.550699	0.032819
1.0	0.000107	0.001386	0.100977	0.550699	0.029681
3.0	0.000107	0.005749	0.02531	0.005214	0.000442
10.0	0.000107	0.029293	0.100977	0.008517	0.069524

H

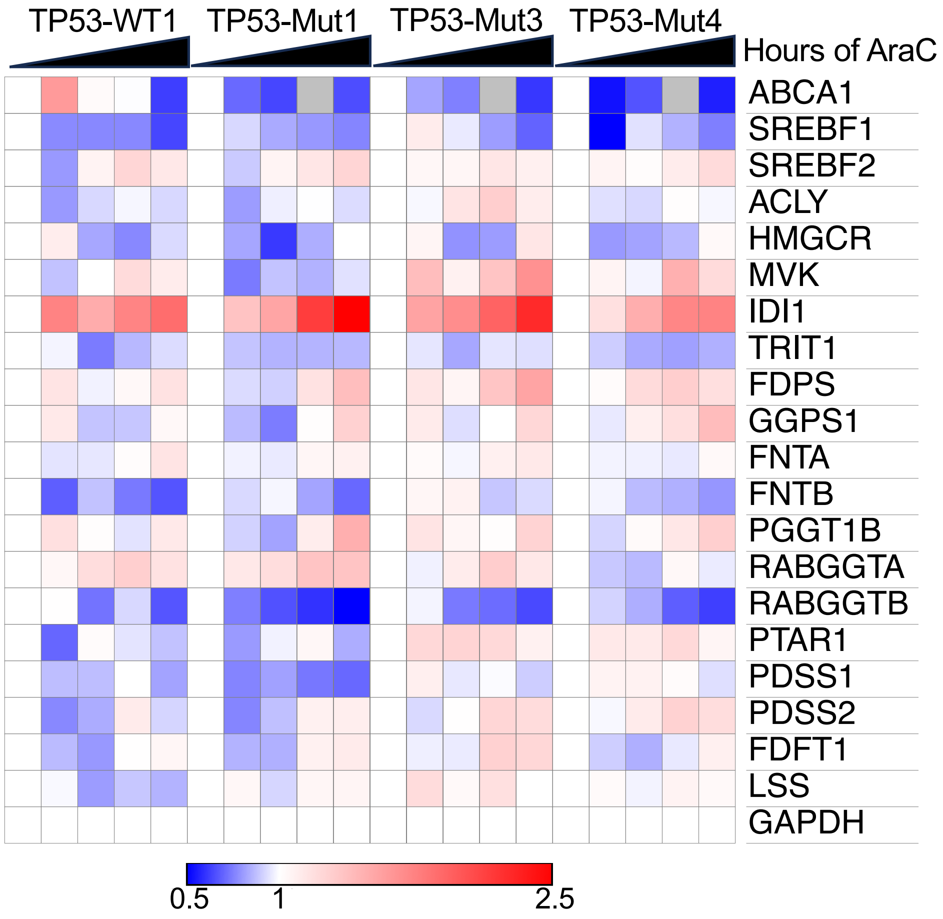

Supplemental Figure 2 Continued:

I

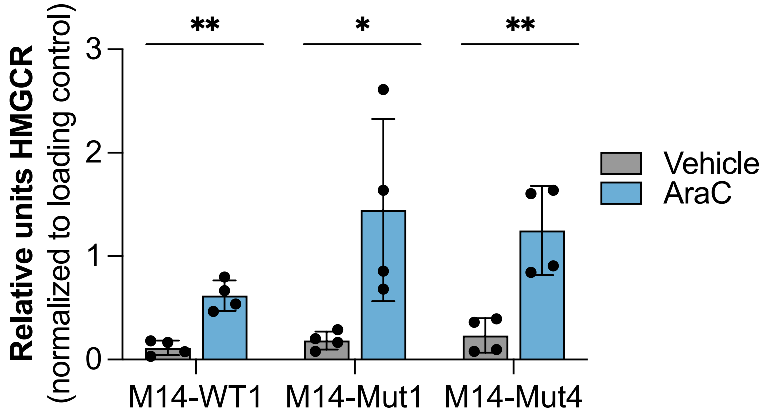

Supplemental Figure 3:

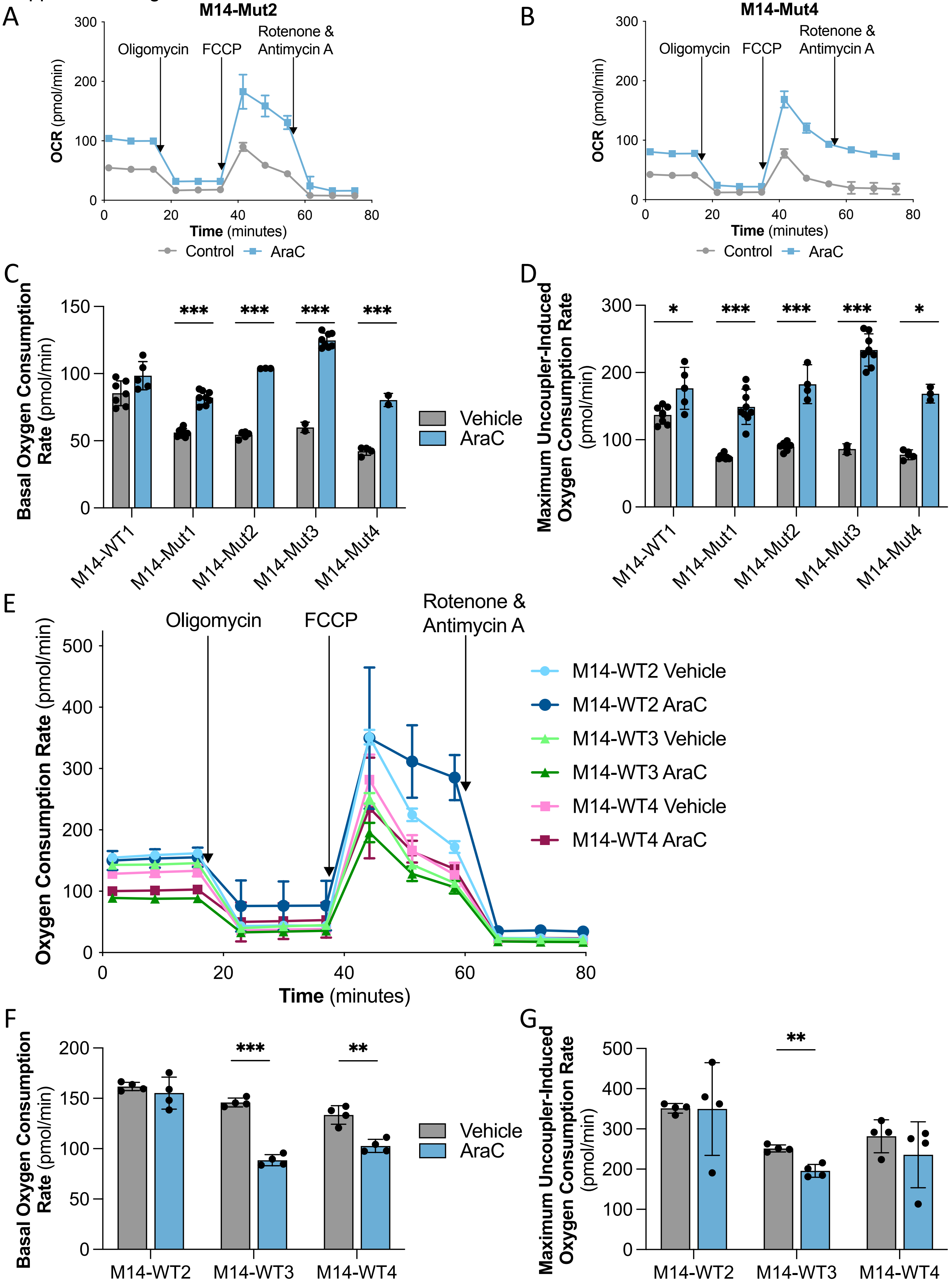

Supplemental Figure 3 Continued:

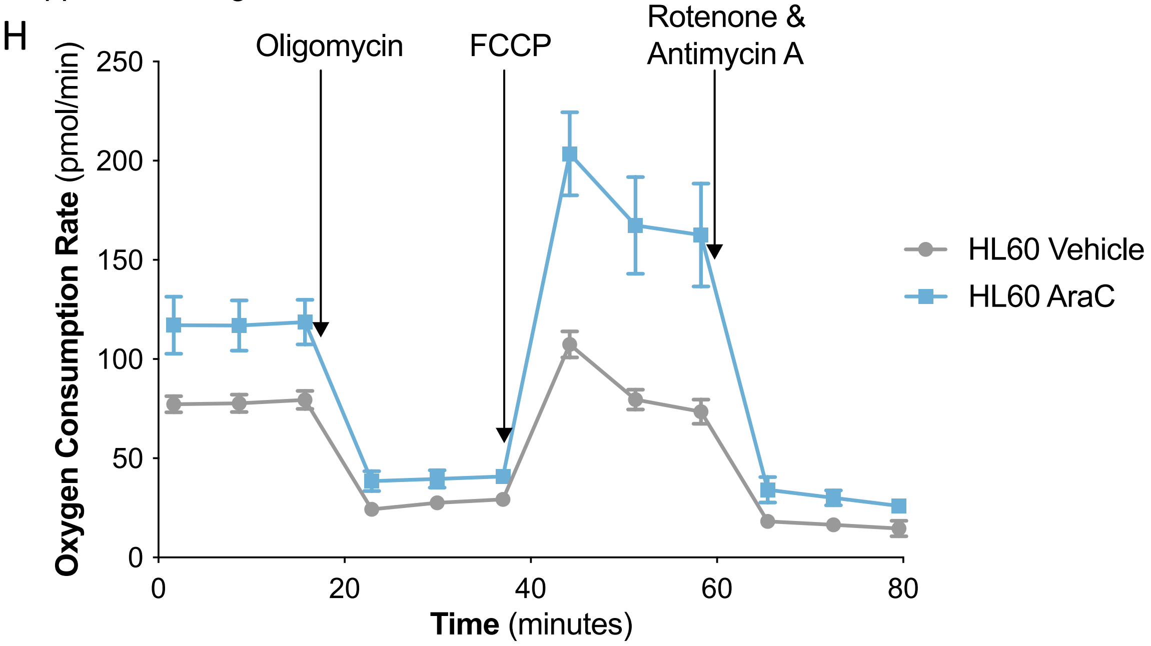

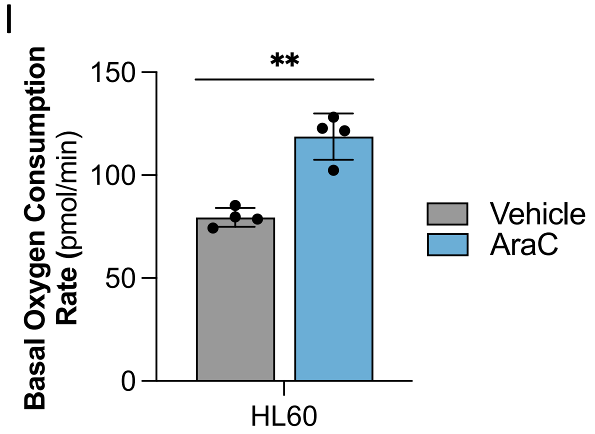

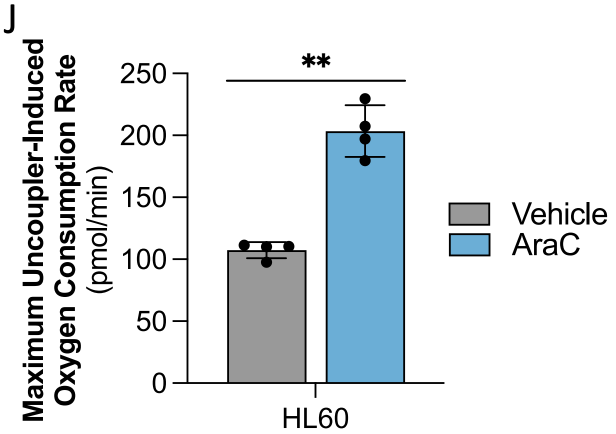

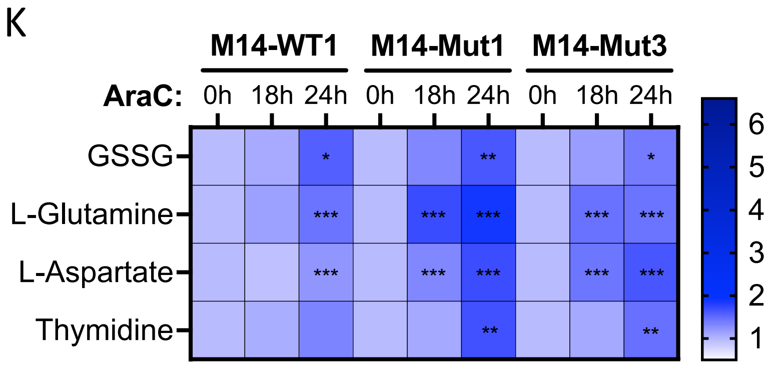

Supplemental Figure 4:

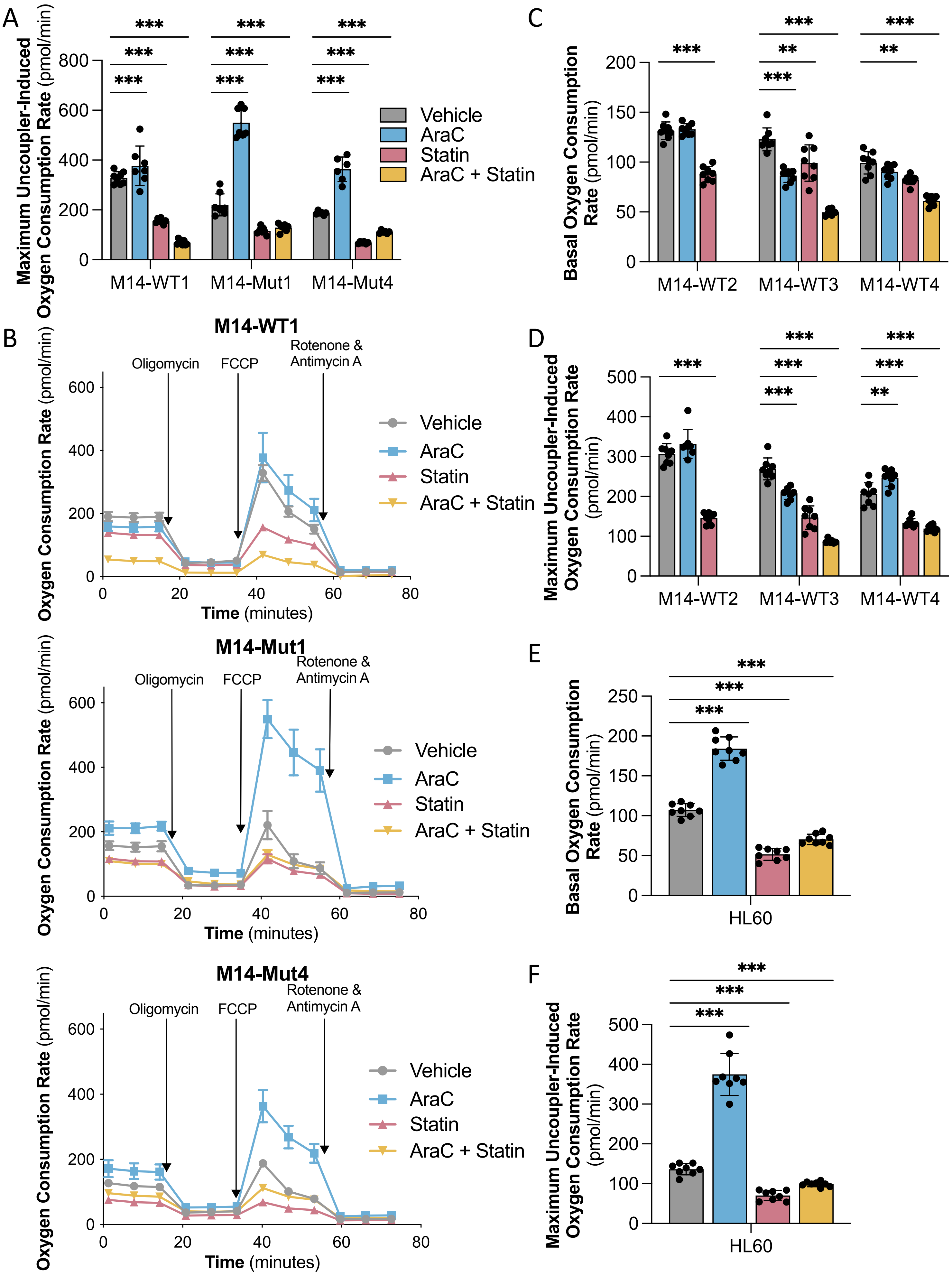

Supplemental Figure 4 Continued:

G

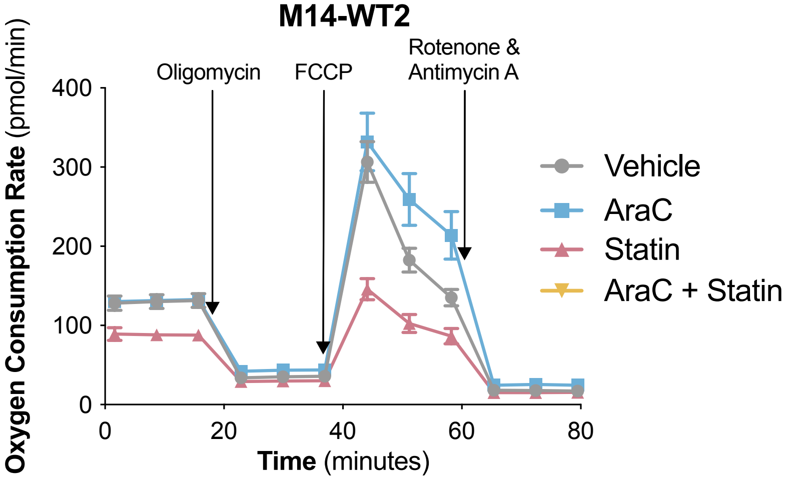

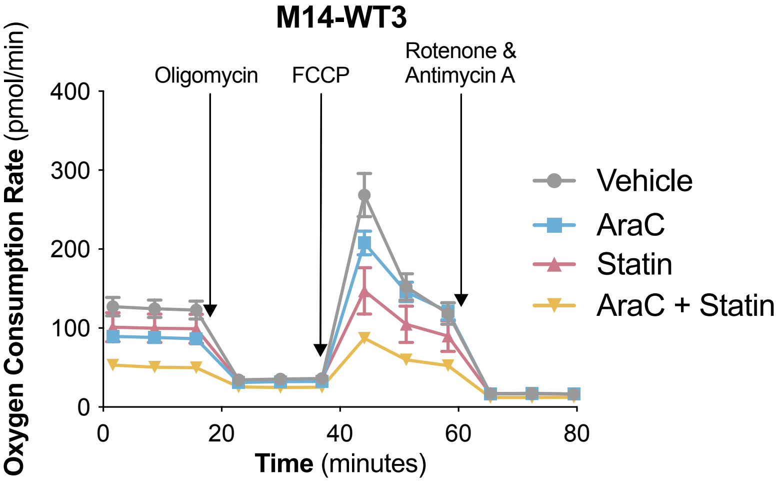

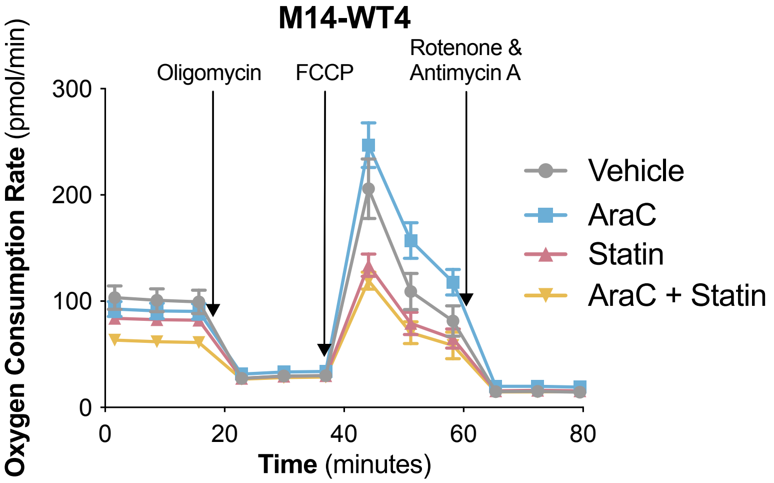

H

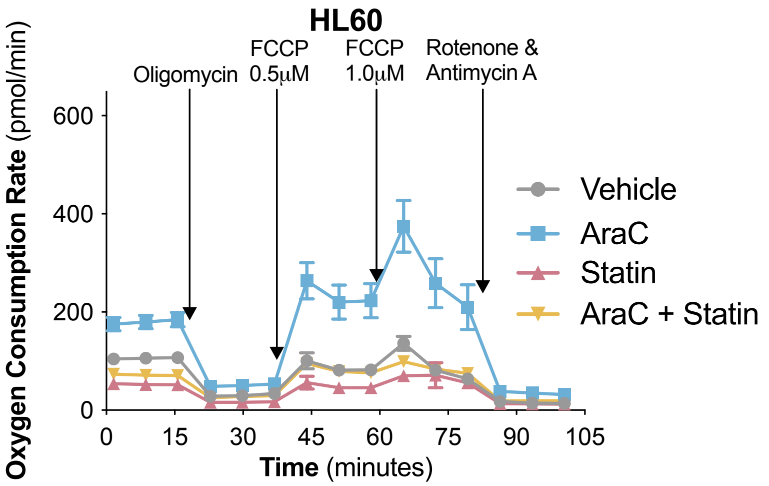

I

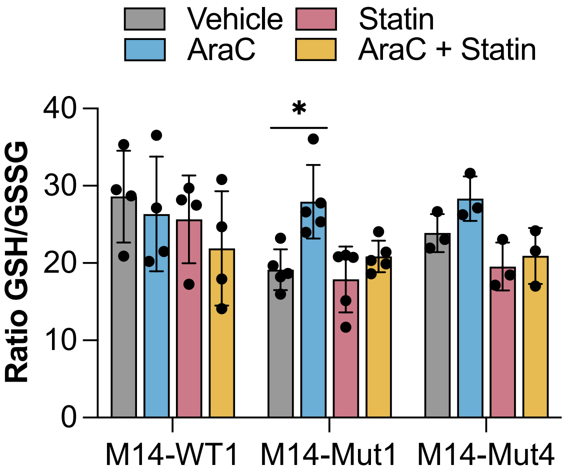

Supplemental Figure 5:

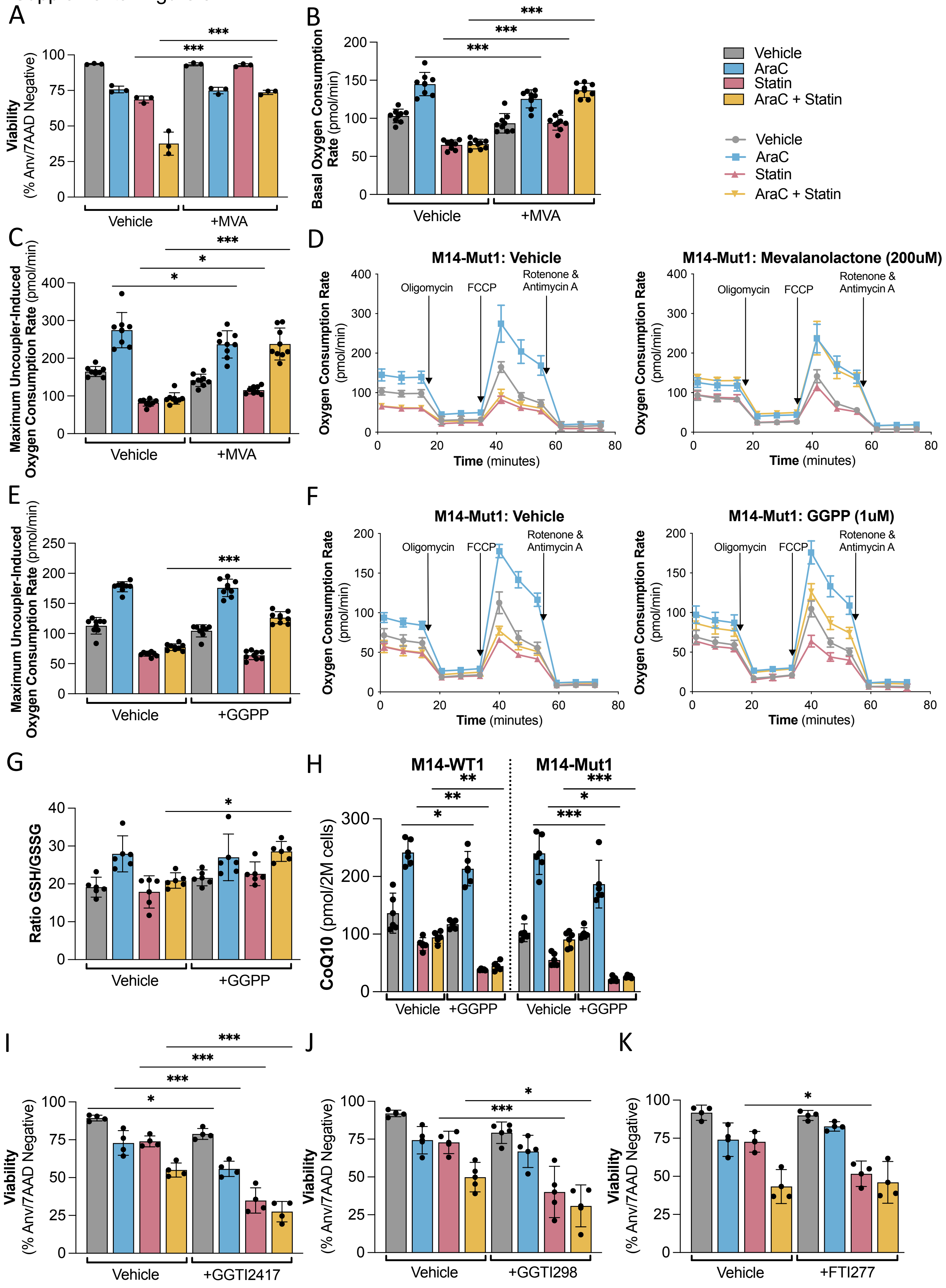

Supplemental Figure 6:

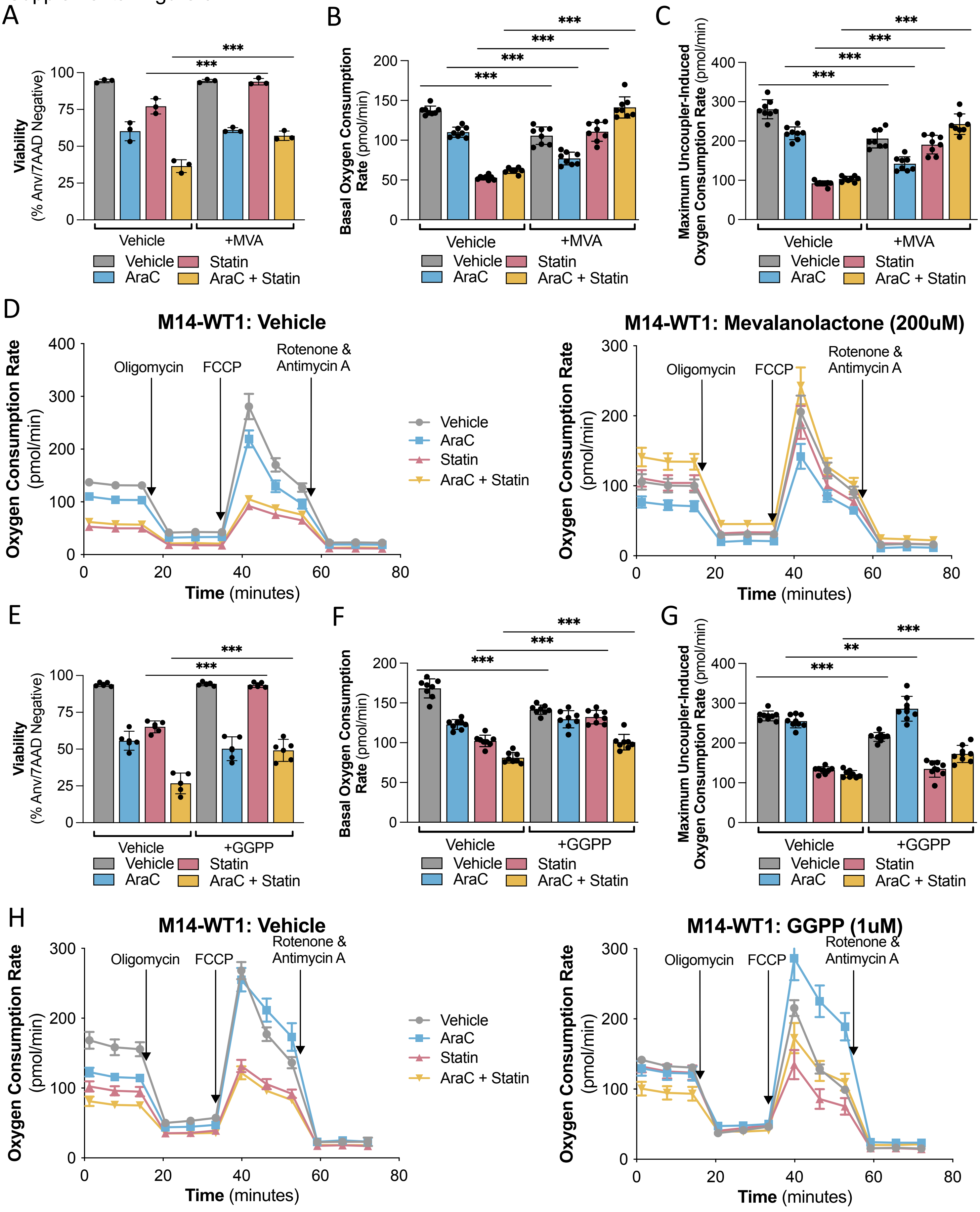

Supplemental Figure 6 Continued:

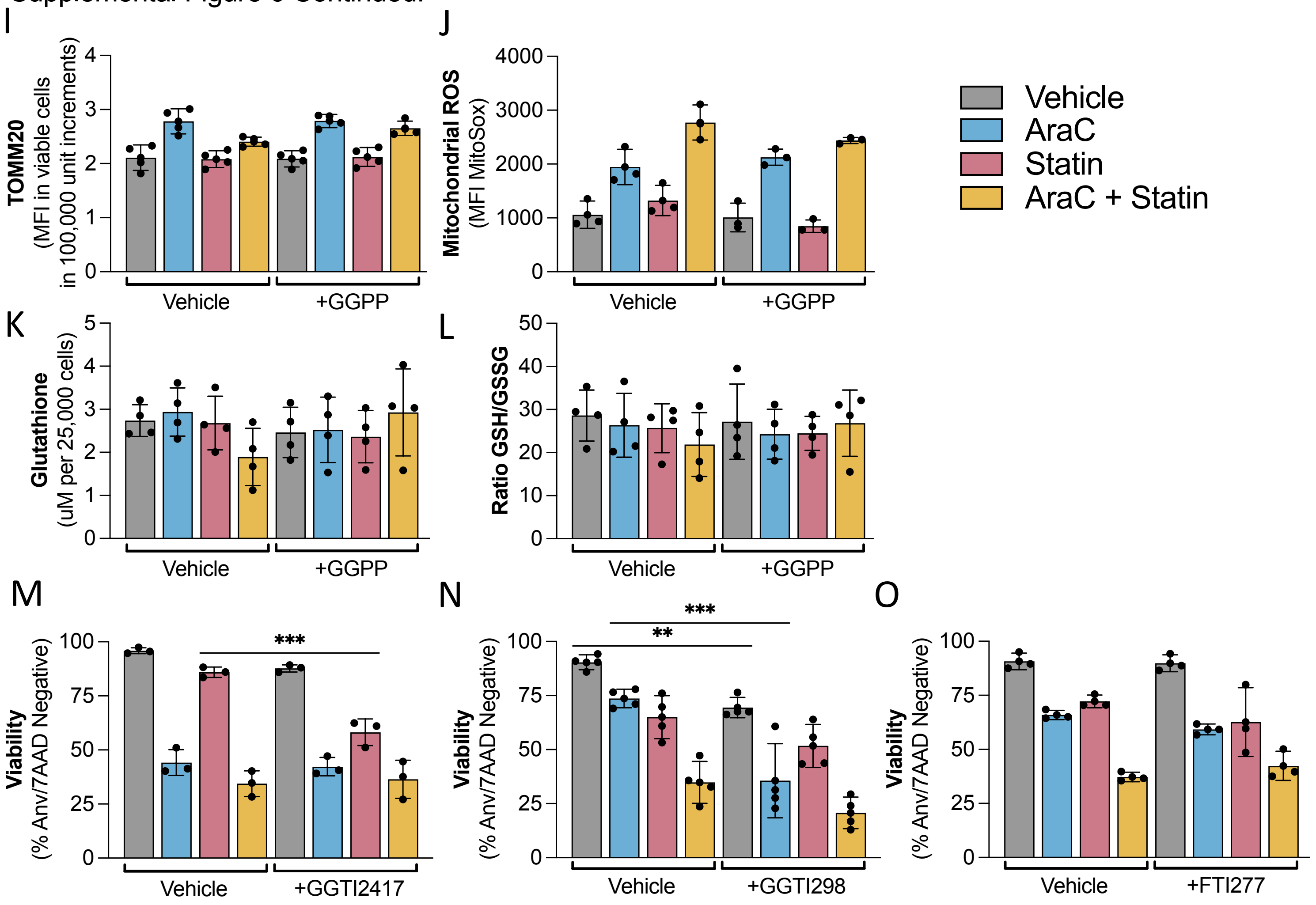

Supplemental Figure 7:

A

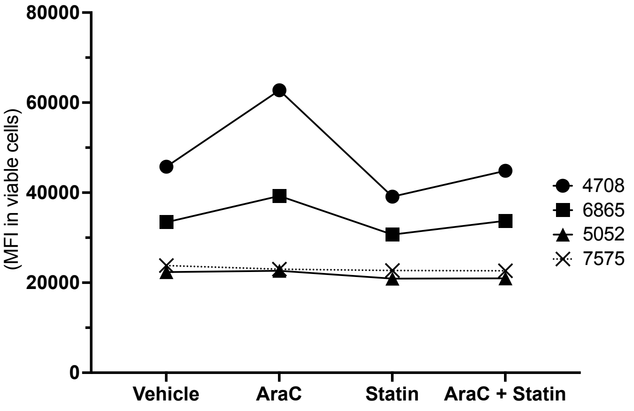

B

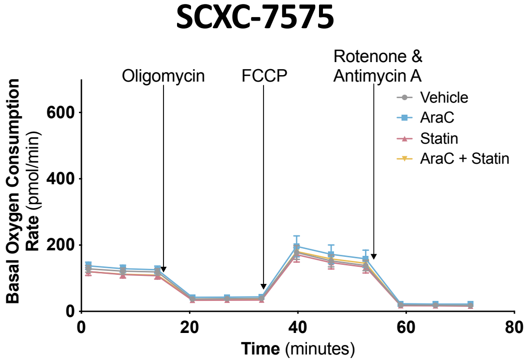

C

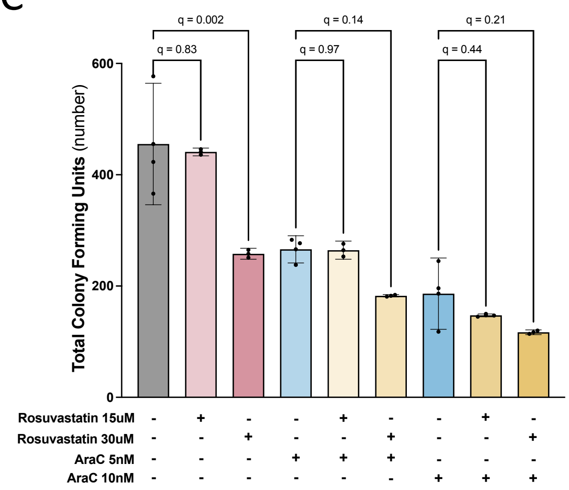

Supplemental Figure 8:

A

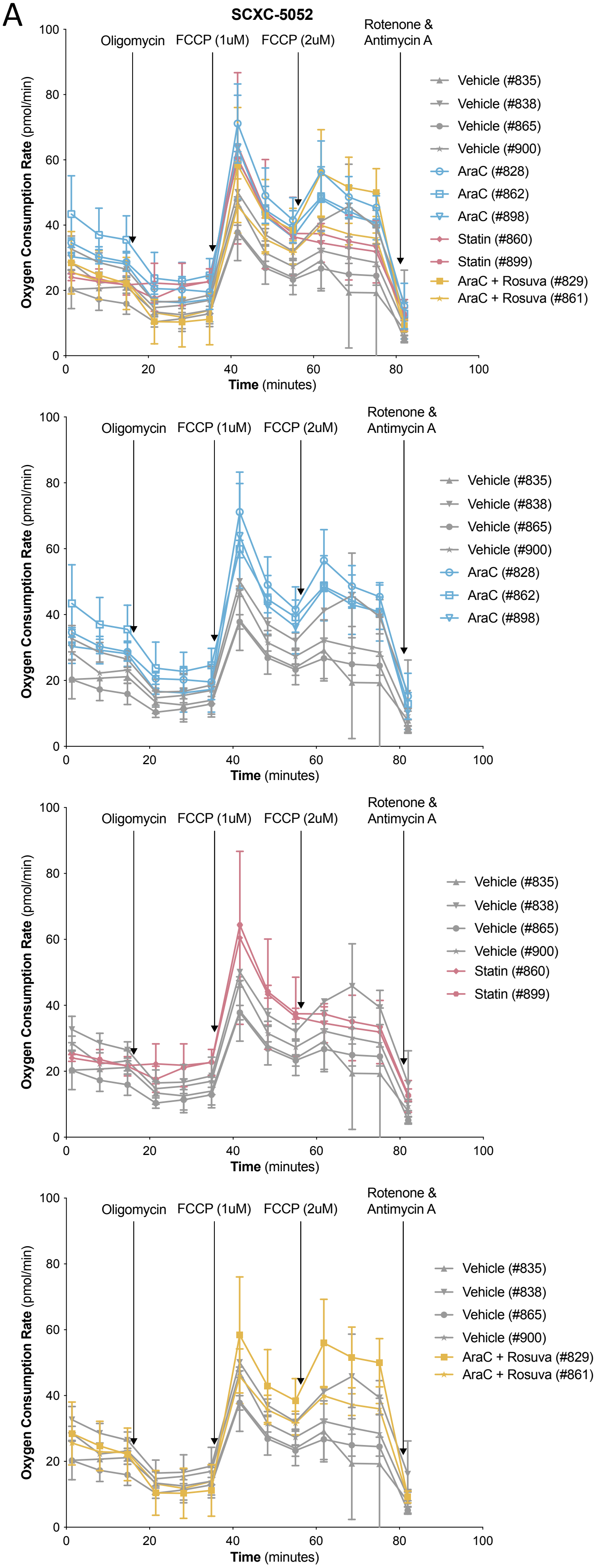

Supplemental Figure 8 Continued:

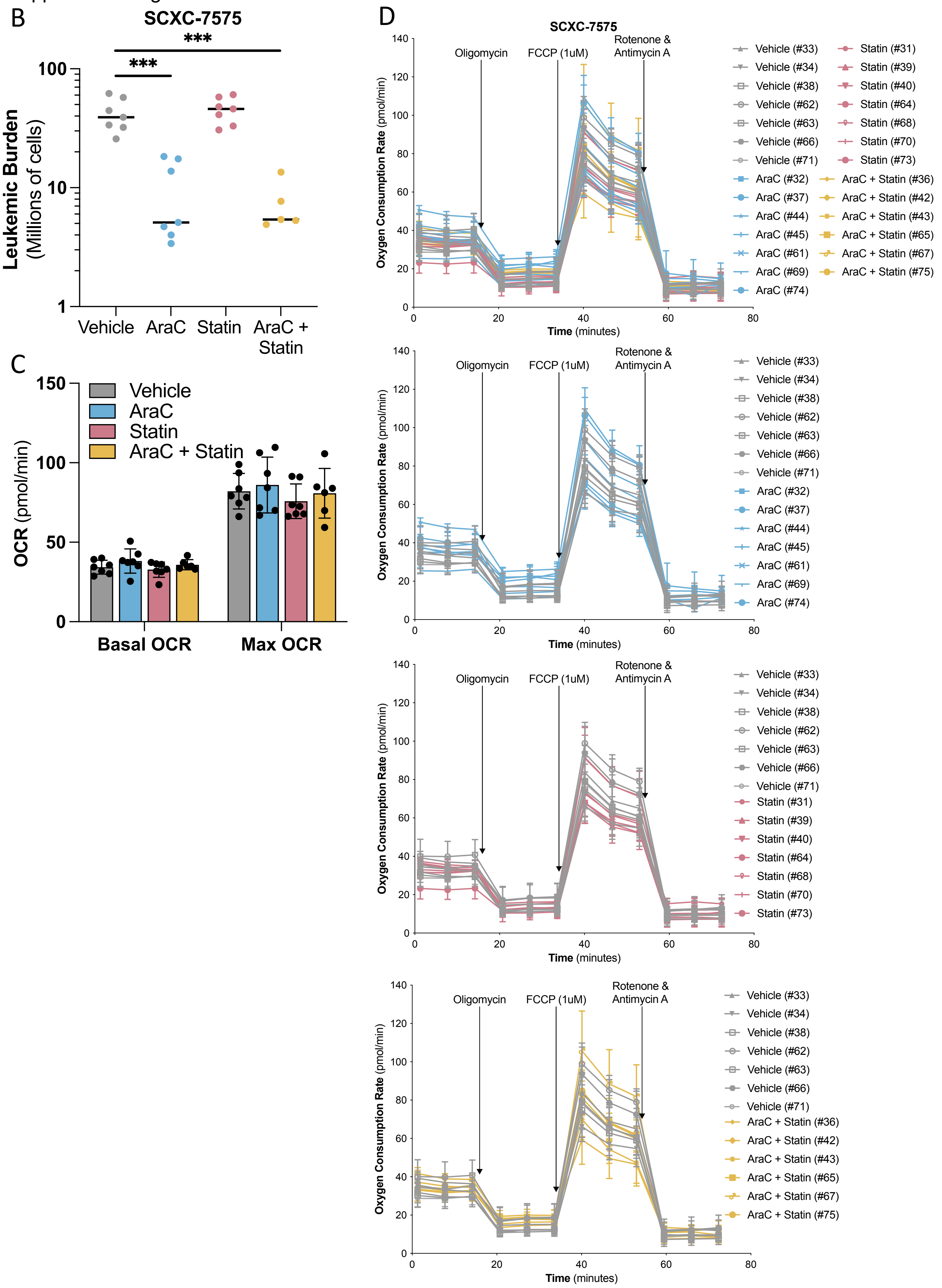

Supplement: Supplementary file 1 — Supplementary Materials [file 41375_2025_2668_MOESM1_ESM.pdf]
